# Size-Exclusion Chromatography–Electrospray-Ionization Mass Spectrometry To Characterize End Group and Chemical Distribution of Poly(lactide-*co*-glycolide) Copolymers

**DOI:** 10.1021/jasms.4c00447

**Published:** 2025-03-31

**Authors:** Masashi Serizawa, Pieter van Delft, Peter J. Schoenmakers, Ron A. H. Peters, Andrea F. G. Gargano

**Affiliations:** a Van’t Hoff Institute for Molecular Sciences, University of Amsterdam, Science Park 904, 1098 XH Amsterdam, The Netherlands; b Centre for Analytical Sciences Amsterdam, Science Park 904, 1098 XH Amsterdam, The Netherlands; c Material Characterization Laboratory, Mitsubishi Chemical Corporation, 1000 Kamoshida-cho, Aoba-ku, Yokohama, Kanagawa 227-8502, Japan; d Corbion, 4200 AA Gorinchem, The Netherlands; e Covestro, TAP, Group Innovation and Sustainibility, Sluisweg 12, 5145 PE Waalwijk, The Netherlands

**Keywords:** size exclusion chromatography−electrospray ionization mass spectrometry (SEC-MS), poly(lactide-*co*-glycolide) (PLGA), chemical-composition distribution, sequence distribution, functionality-type distribution

## Abstract

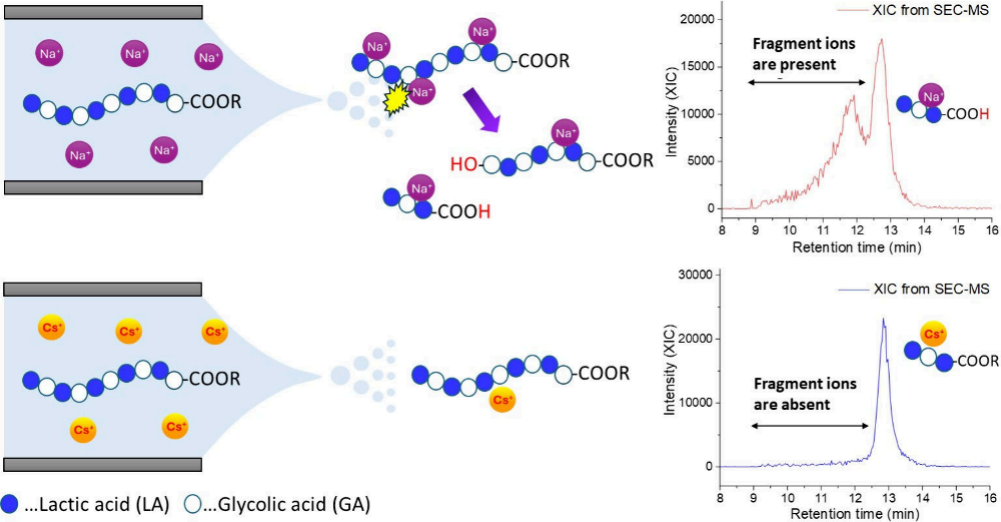

The characterization of the microstructure of *in vivo* degradable polyesters is gaining increased interest thanks to their high-performance applications, such as drug delivery systems. The design of such material requires a high level of understanding of the critical material attributes of the polyesters, such as molecular-weight distribution (MWD), chemical-composition distribution (CCD), and end-groups (functionality-type distribution, FTD). Size-exclusion chromatography (SEC) hyphenated with mass spectrometry (MS) is an effective method for analyzing the microstructure of polymers. While the MWD can be determined by size-exclusion chromatography hyphenated with ultraviolet spectrometry and refractive index, the CCD and FTD can be determined by SEC-MS. However, previous applications of SEC-MS have not assessed if polymer fragmentation can occur during the analysis process. In order to correctly interpret CCD and FTD, it is important to establish whether SEC-MS methods can be applied to biodegradable polymers and to recognize if fragmentation processes occur. In this study, we investigate whether SEC-MS methods can be applied to PLGA biodegradable polyesters. The research demonstrates that the choice of alkali metal salt used during ionization can influence the stability of PLGA during SEC-MS analysis. CsI was found to minimize fragmentations during ESI-MS, simplifying the MS spectra and allowing isomeric PLGA structures to be distinguished. The resulting method facilitates FTD and CCD determination. Additionally, when combined with selective degradation, the described method can provide insights into the “blockiness” of the polymer and support the development of sequence-controlled PLGA synthesis.

## 1 Introduction

There is an increased interest in characterizing the microstructure of biodegradable polyesters used in various applications, such as packaging, regenerative implants, and sustained drug delivery systems.^1−8^ In sustained drug delivery formulations, it is particularly important to control the speed of the polymer-degradation process because this relates to the rate of drug release. An important example of biodegradable biobased polymers is poly(lactide-*co*-glycolide, PLGA), which can be broken down into smaller oligomers and monomers that can be easily excreted. Because of its degradability and biocompatibility, PLGA has attracted significant attention for applications such as tissue engineering and drug delivery systems.^2,7,9^

The intended purpose determines the desired properties of PLGA, and these can, in turn, be related to the chemical structure.^10^ For instance, previous research has shown that the molecular weight, chemical composition, and end group of the polymer will affect the size of PLGA nanoparticles, the amount of drug that can be loaded into these, and the rate at which they biodegrade.^9,11−13^

Therefore, knowledge of the molecular-weight distribution (MWD), chemical-composition distribution (CCD), and functionality-type (end-group) distribution (FTD) is crucial for creating tailored high-performance polyesters. Size-exclusion chromatography (SEC) coupled with ultraviolet spectrometry (UV)/refractive index (RI) detectors and electrospray-ionization mass spectrometry (ESI-MS) is a powerful analytical tool for polymer characterization.^14^ SEC allows obtaining MWDs of polymers. UV or RI detection is used to obtain abundance profiles that can be transformed into MWDs, while accurate mass analysis by ESI-MS detection allows the identification of the molecular structures and the determination of CCD and FTD.^14,15^

Jovic et al. reported several practical advantages of the SEC-MS, including improved signal intensities when compared with ESI-MS with direct infusion, effective estimation of end-group masses, and determination of the absolute MWD.^14^ SEC-MS has been applied to a variety of polymers, including polymethyl methacrylate (PMMA), polyacrylamide (PAM), polyethylene glycol (PEG), and aromatic polyesters.^16−20^

Thus, SEC-MS is an excellent method for simultaneously determining MWDs, CCDs, and FTDs.

However, the applications reported so far have mainly focused on stable polymers, which are unlikely to fragment during the ESI-MS process. In the analysis of biodegradable polyesters, it is critical to assess whether fragmentation of the polymers occurs during ESI-MS. Fragment ions may have different end groups and chemical compositions, altering the resulting FTD and CCD. ESI-MS is generally considered a soft ionization method, but in-source fragmentation has been reported in some cases.^21,22^ Therefore, when characterizing biodegradable polymers, it is important to determine whether a detected ion peak contains fragment ions in the gas phase and to select analytical conditions to avoid fragmentation. Notably, controlling fragmentations in the ESI-MS process is crucial for applications such as LC-MS/MS, ion mobility MS, and other LC-MS-based methods.^20,23−25^

Polyester end-groups are often carboxylic acid, alkyl, or hydroxyl groups—originating from the monomers of the acid or the alcohol repeating units used during polycondensation or from alkyl alcohol initiators used during open-ring polymerization, as opposed to initiators or other distinct chemistries (e.g., AIBN) in radical polymerization. Therefore, it is important to assess whether, for example, acid-terminated polyesters and hydroxyl-terminated polyesters are the original polymers in the sample or whether they are fragment ions created during analysis. To address this issue, in our work, we examined SEC-MS analysis of polymers with a low number of repeating units (5–20) to determine whether the ion peak detected by SEC-MS represented molecular or fragment ions. In contrast to molecular ions, ions occurring due to fragmentation do not exhibit a single, approximately Gaussian distribution in SEC but occur across a broad range of masses. Based on this premise, we set out to investigate different parameters that may lead to polymer fragmentation, including the type of alkali metal used in the makeup solution and the instrumental conditions(capillary voltage, trap-cone voltage, sampling cone voltage, desolvation temperature, source temperature, and gas pressure). We also aimed to characterize the lactic acid-rich sequence in PLGA after chemical hydrolysis.

## 2 Experimental Section

### 2.1 Chemicals and Samples

For SEC-MS, the solvents used for separation is tetrahydrofuran (THF) (unstabilized, GPC grade) from Biosolve (Valkenswaard, The Netherlands) and formic acid (>98%, puriss p.p. grade) from Sigma-Aldrich (Darmstadt, Germany).

The make-up flow used for mass spectrometry included acetonitrile (ACN), methanol (MeOH), and ultrapure water, all obtained from Biosolve. Lithium iodide (LiI), sodium iodide (NaI), potassium iodide (KI), rubidium iodide (RbI), Cesium iodide (CsI), 3-nitrobenzyl alcohol (NBA), and triethylamine (TEA) were used as ionization agents with purities exceeding 99.5%(w/w) and obtained from Sigma-Aldrich. Polystyrene (PSt) standards to calibrate the SEC separation were obtained from Polymer Laboratories (Shropshire, UK). NMR solvents, including deuterated chloroform (chloroform-*D*_*1*_), were obtained from Sigma-Aldrich.

PLA and PLGA used throughout this study are commercially available from Akina. as e-L100-S and e-L50-S, respectively. Acid-terminated PLGA, a-L50-S, was kindly provided by Corbion (Gorinchem, The Netherlands). PNI was synthesized from neopentyl glycol (NPG) and isophthalic acid (IPA). The same sample was used by Groeneveld et al.^26^Table 1 summarizes the samples used throughout this study.

**Table 1:** Abbreviations, End Group Compositions, Polystyrene-Equivalent Molar Masses, and Polydispersity of the Investigated Polyesters^a^

			*M*_n_ (kDa)^b^	*M*_w_ (kDa)^b^	PDI^b^
Abbreviation	End-group	Chemistry	Mean ± SD	Mean ± SD	Mean ± SD
e-L100-S^c^	Alkyl/Alcohol	LA = 100	23.2 ± 0.5	67.1 ± 2.6	2.9 ± 0.2
e-L50-S	Alkyl/Alcohol	LA/GA = 50/50	12.1 ± 0.2	28.7 ± 4.0	2.4 ± 0.4
a-L50-S	Acid/Alcohol	LA/GA = 50/50	10.9 ± 0.4	37.7 ± 0.5	3.5 ± 0.4
PNI	Diol	NPG/IPA	7.1 ± 0.1	17.0 ± 0.5	2.4 ± 0.0

a For chromatographic conditions, see the Experimental Section.

b SEC-UV chromatograms are shown in Supporting Information (Figure S6 and S7). SD (standard deviation) of the mean for three sample measurements.

c e-L100-S is poly(d,l-lactide).

**Figure 1:**
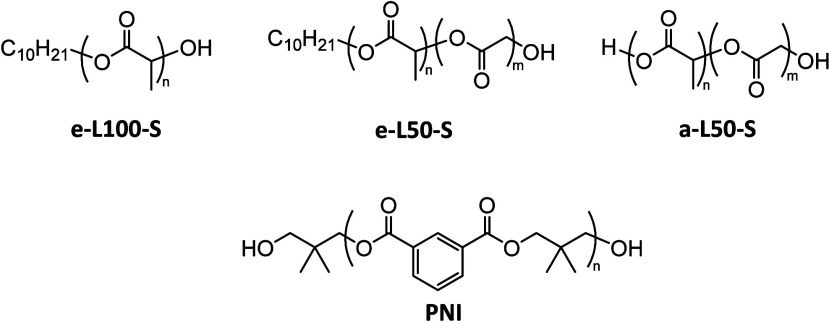
Structures of the monomers composing the polymers selected for this study.

The structures of the polymers, as well as molecular weight and other characteristics, are reported in Figure 1 and Table 1. In our discussion, the aliphatic-ester and free-carboxylic acid end groups, as shown in Figure 1, will be referred to as alkyl and acid end groups, respectively.

The polymer end-groups were confirmed in the ^1^H NMR experiments and normal phase liquid chromatography (NPLC), as depicted in Figures S1–S5. The acid-terminated PLGA, a-L50-S, exhibited a peak at about 2.0 ppm, indicating water. Conversely, the alkyl-terminated PLGAs, e-L100-S and a-L50-S, displayed peaks at 0.8 and 1.3 ppm, indicative of an alkyl-end group, with no peaks representing at about 2.0 ppm (Figures S1 and S2 respectively). These results confirm that e-L100-S and e-L50-S are primarily alkyl-terminated PLGAs with minimal acid-terminated PLGAs.

Furthermore, a NPLC method was used to characterize the end-group distribution.^27^ This approach allowed us to separate acid-terminated and non-acid-terminated PLGA, as illustrated in Figure S5. The amounts of acid-terminated PL(G)A in the samples were as follows: e-L100-S contained under 0.1 area%, e-L50-S contained under 0.1 area%, and a-L50-S had over 99.9 area%.

To perform chemical degradation, 5.0 mg of e-L50-S was dissolved in 0.8 mL of ACN and mixed with 180 μL of water and 20 μL of TEA. The chemical degradation was performed by heating under continuous shaking in a thermal shaker for 0.5 h at 60 °C and 400 rpm. After chemical degradation, 200 μL of the solution were transferred to a vial and dried overnight (at room temperature under N_2_ stream). The dried sample was redissolved in THF and used for SEC-MS analysis. These degradation conditions were modified based on a previous report by Pourasghar et al.^28^

### 2.2 Instrumentation and HPLC Columns

SEC-UV/MS experiments were performed using a Waters Acquity UPLC H-Class system (Waters, Milford, MA, USA) consisting of a binary solvent manager, sample manager, column manager, and UV detector connected to a Waters Synapt G2 high-resolution mass spectrometer. An Agilent PLgel MesoPore column (250 × 2.1 mm i.d., 3.0 μm particles) was used for SEC.

NPLC experiments were performed using a Waters Acquity UPLC H-Class system (Waters, Milford, MA, USA) consisting of a quaternary solvent manager, sample manager, column manager, and ELS detector. A Phenomenex Luna HILIC (150 × 2.0 mm i.d., 3.0 μm particles) column was used as the NPLC column.

### 2.3 Analytical Conditions

#### 2.3.1 SEC-UV/MS

**Figure 2:**
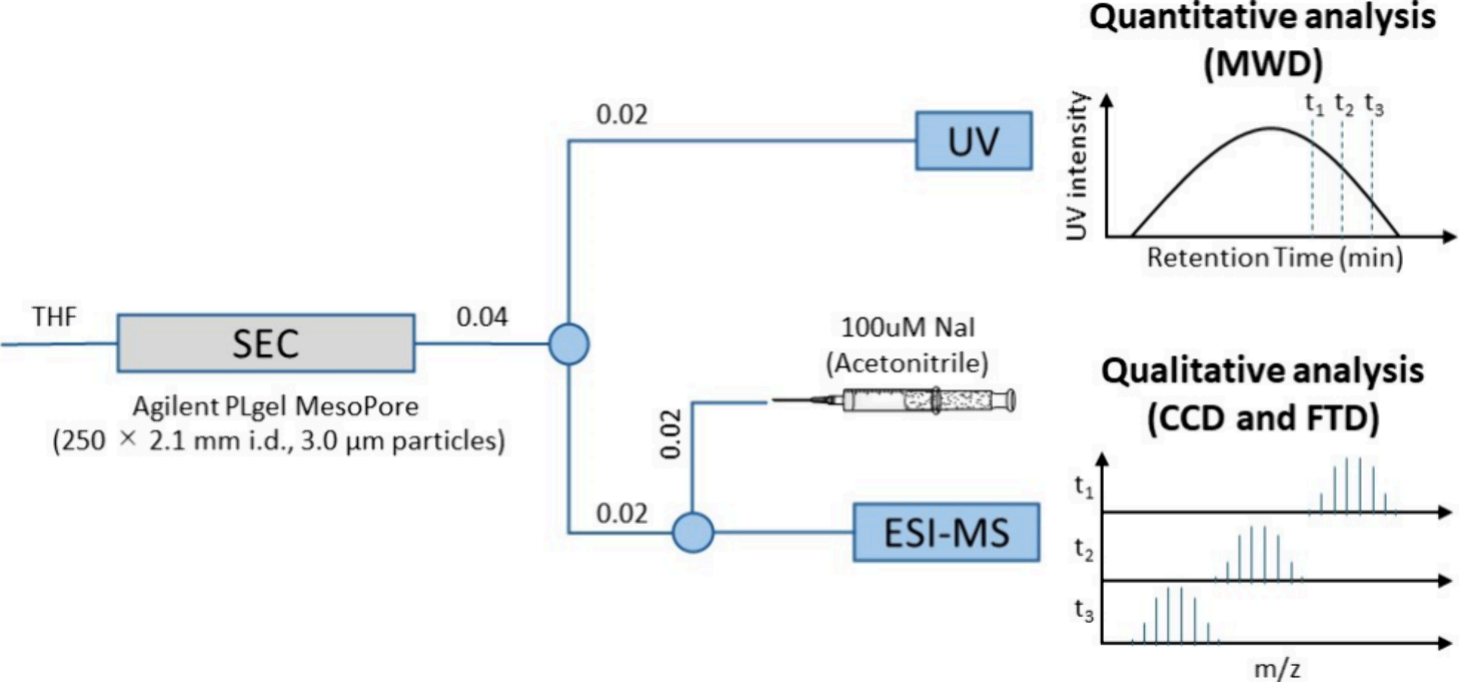
Schematic representation of the SEC-MS/UV setup and the information that is extracted from the UV and ESI-MS detector.^17^ Numbers indicate flow rates in mL·min^–1^.

A schematic illustration of the setup is presented in Figure 2. UV detection at 220 nm using a small flow cell (V_det_ = 500 nL) was used to detect the PLA and PLGA polymers. To reduce the dead volumes between the exit of the SEC column and the entrance of the MS, the flow was split postcolumn and the UV detector was placed parallel to the MS. To perform ESI-MS, a makeup flow was added to introduce the ionization agent that allow for polymer ionization (Figure 2).

For the SEC–UV/MS experiments, 5.0 μL of solute was injected into the SEC column thermostated at a temperature of 23 °C and operated at a flow rate of 40 μL/min. THF containing 0.1% (v/v) formic acid was used as the mobile phase. Column calibration and estimation of *M*_n_ and *M*_w_ were based on polystyrene standards (M_p_ ranging from 679.0 kDa, 382.0 kDa, 114.2 kDa, 29.3 kDa, 10.0 kDa, 7.0 kDa, 3.3 kDa, 1.7 kDa, to 0.6 kDa). For parallel UV/MS detection, the SEC effluent was split using a T-piece and restriction capillaries (450 × 0.075 mm i.d.), and the effluent was split 1:1 to the UV and MS detector, respectively. At the diverter valve of the mass spectrometer, a makeup flow containing the ionization agent(s) was infused to create a total flow to the ESI inlet of 40 μL/min, with a 1:1 ratio of analytical and makeup flow.

To detect a variety of polyesters with different chemical structures, SEC-UV/MS was conducted in positive ESI mode rather than in negative ESI mode, which primarily detects deprotonated polymers due to potentially including the risk of differing ionization efficiencies between acid-terminated and non-acid-terminated polymers in negative ESI mode.^29^

MS conditions applied: *m*/*z* range, 200–5000; scan time, 1 s; positive ESI; time-of-flight MS resolution mode; capillary voltage, 3.5 kV; sampling cone, 200 V; trap collision energy, 10 eV; source temperature 120 °C; desolvation temperature, 350 °C; nitrogen desolvation gas flow, 850 L/h; nebulizer gas flow, 100 L/h. Mass calibration was performed using NaI as reference mass.

The UV detector was set at a wavelength of 220 nm with a bandwidth of 4.8 nm. The photodiode detector was set to a scan rate of 20 Hz.

0.1 mM alkali metal salt (LiI, NaI, KI, RbI, or CsI) in a solution of 10% water and 90% ACN was used as a makeup flow for studying the influence of alkali metal salts. In addition, a supercharging agent, 0.5% v/v NBA in 0.1 mM NaI in a solution of 10% water and 90% ACN, and a charge-reduction agent, 1.2 mM TEA in a solution of 10% water and 90% ACN, were tested as makeup flow.^15,24,26^

Instrument control, data acquisition, and data processing of all experiments were performed using MassLynx 4.1 (Waters).

#### 2.3.2 ^1^H NMR

^1^H NMR measurements were performed in chloroform-*D*_*1*_ on a Bruker (Rheinstetten, Germany) Avance II 300-MHz NMR spectrometer at 298 K. Approximately 2 mg of each sample were dissolved in 0.5 mL chloroform-*D*_*1*_. A total of 16 scans were recorded for the ^1^H measurements with a relaxation delay of 1.0 s and a flip angle of 30°. Mnova 15.0.1 software was used to interpret the ^1^H NMR spectra.

#### 2.3.3 NPLC

For the NPLC experiments, 1.0 μL of solute was injected and the separation was performed at a flow rate of 200 μL/min and 55 °C. *n*-hexane, ethyl acetate containing 0.1% (v/v) triethylamine, and THF containing 0.1% (v/v) formic acid were used as the mobile phase, employing the NPLC ternary gradient method described in literature.^27^

### 2.4 SEC-UV/MS Data Analysis

The SEC-UV/MS data were acquired using MassLynx software (Waters, version 4.1).

Origin 2019b (Origin Lab, Massachusetts, USA) software was used to visualize size exclusion chromatograms, mass spectra, and heat maps (contour color full). For visualization of size-exclusion chromatograms using UV detection, the raw data was smoothed using a Savitzky-Golay finite impulse response (FIR) smoothing filter of third polynomial order and a window of 100 points.

To obtain the number-average molar mass (*M*_n_), mass-average molar mass (*M*_w_), polydispersity index (PDI), and extracted-SEC curves from individual distributions present in the SEC-UV data, regions of interest were defined, extracted, and summed on the ^2^D axis. The time axis was then transformed to molecular weight according to the SEC calibration, and the raw data were used to determine *M*_n_, *M*_w_, and PDI.

Peak lists derived from chromatograms and mass spectra were extracted from MassLynx 4.1. Data were exported as CSV files. MSPolyCalc was used to identify detected peaks in mass spectra.^30^

MSPolyCalc analytical conditions for PLGAs were: charge range; min 1 and max 5, range of *m*/*z*; min 200 and max 5000, mass tolerance, 15 ppm; threshold (peak picking), 0.1; threshold (similarly); 90%, width; bottom 0.2 and top 0.1; zone; low −0.5 and high 0.5; Relative intensity; over 15%.

The following equation was used for the calculation of the average charge state (ACS):^15^
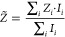
1where *Z̃* is the intensity-weighted ACS, *Z*_*i*_ is the charge state of each peak i, and *I*_*i*_ is the intensity of each peak obtained from MSPolyCalc.^30^

The following equation was used for the calculation of the proportions of alkyl and acid-terminated polymers:
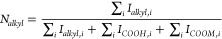
2
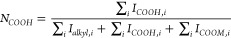
3
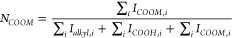
4where *N*_*alkyl*_ is the proportion of alkyl-terminated PLGA (alkyl-PLGA), with α and ω end-groups C_10_H_21_ and OH; *N*_*COOM*_ is the proportion of acid-terminated PLGA(acid-PLGA), with α and ω end-groups H and OH; *N*_*COOM*_ is the proportion of alkali metal carboxylated-terminated PLGA, with α and ω end-groups are M, which is the alkali metal used in the SEC-MS analysis, and OH.

Cyclic or alkene-terminated PLGA, generated in the mechanism described in Figure S8, were also detected but are not included in our end-group calculations as these components are known to be a minor component of the initial material.^31^ Excluding cyclic end groups from our analysis introduces a potential source of error because their omission leads to an overestimation of the true end-group concentration.

Moreover, in eqs 1 through 4 it is assumed that the MS response is equal for all peaks. However, the ionization efficiency and detection sensitivity in mass spectrometry can vary depending on the molecular weight, chemical structure, and end-group functionality of the polymer chains. This variability can lead to biases in the calculated end-group concentrations.

To simplify our calculations, we assumed equal response factors for all peaks. Therefore, the metrics *N*_*COOH*_ and *N*_*COOM*_ have to be considered qualitative indications of the presence or absence of acidic end groups, as the response factors between end groups are different. This is also confirmed by experiments conducted on PLGA acid and ester-terminated polymers with similar molecular weight distribution analyzed individually and as a 1:1 mixture (section 3.4). A lower response for acid-terminated polymers (46% of the ester-terminated) was observed here. Therefore, we expect an underestimation of acid groups in our measurements.

## 3 Results and Discussion

Our investigation was aimed at characterizing the MWD, CCD, and FTD of biodegradable polyesters based on polylactic acid. These characteristics are closely related to the properties of the polymer, such as degradability, solvent solubility, glass-transition point (*T*_*g*_), and other polymer properties.^27^

To achieve our goals, we systematically studied the ESI conditions, particularly the composition of the makeup flow and its influence on the end group functionality observed in mass spectrometry experiments.

### 3.1 Size-Exclusion Chromatography–High-Resolution Mass Spectrometry for the Analysis of PLA and PLGA Polymers

The coupling of size-exclusion chromatography with mass spectrometry extends the range of molecular weights of polymers that can be ionized using ESI-MS. This is often done in combination with optical detection to determine the MWD. In SEC of polymers, mobile phases with flows between 0.2 and 0.8 mL/min are often used, of which only 10 and 20 μL/min are used for MS detection.^16−19,32^ The remaining part is subjected to optical detection.

Here, we adopted a 2.1 mm ID column to perform SEC-UV/MS of PLA and PLGA polymers. This column was designed to characterize low-molecular-weight polymers and presented an approximately linear (logarithmic) calibration curve when analyzing polystyrene polymer standards of *M*_w_ between 29.3 and 0.6 kDa (Figures S6 and S7). This range allowed us to obtain good resolution for polyesters of low molecular weight. The smaller diameter of the column allowed its operation at 40 μL/min, using a linear velocity suitable for SEC (0.2 mm/s, assuming a porosity of 60%). The flow rate reduces solvent consumption and toxic waste. The advantage of reducing solvent consumption can be quantified with greenness metrics, such as the analytical method greenness score (AMGS).^33^ The flow-rate reduction allowed us to significantly improve the Greenness Score, reducing it from 270 to 143, compared to the same method run using a conventional column diameter of 4.6 mm ID while maintaining the same linear velocity (data reported in Table S1).

### 3.2 In-Source Fragmentation of Alkyl-Terminated PLA during SEC-MS Using NaI

Despite ESI-MS being considered a soft ionization method characterized by low in-source fragmentation, it is important to assess whether analytes can undergo fragmentation during the ionization.^34^ This is particularly critical for determining the end-groups of biodegradable PLA and PLGA samples. For SEC-MS analysis of polymers such as polyesters, sodium iodide is often used as an ionization agent.^14,26^ We hypothesized that the type of alkali-metal salt used strongly influences the end-group intensities observed by mass spectrometry. We investigated these effects by changing the composition of the additional (“make-up”) flow used to mix the ionization agent after the SEC separation. The makeup flow was composed of 0.1 mM lithium, sodium, potassium, rubidium, or cesium iodide, used as an ionization agent, in a solution of 10% water and 90% ACN.

**Figure 3:**
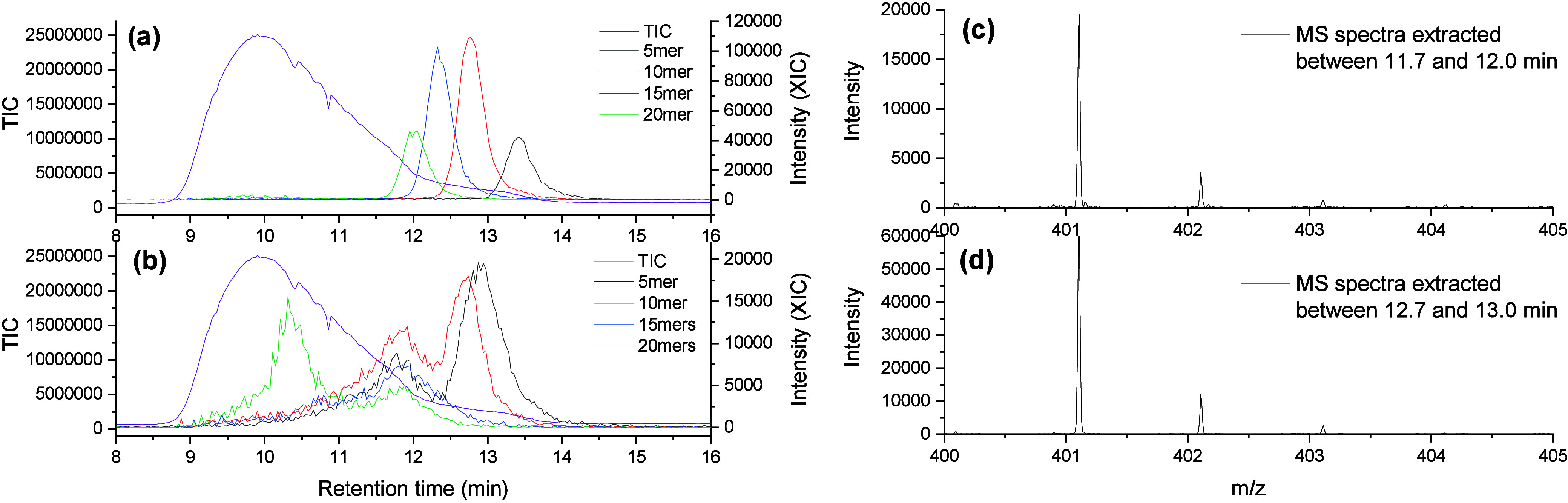
SEC-MS analysis using NaI as a cation additive of e-L100-S. TIC and XICs of oligomers (5–20 repeating units) with the alkyl (a) and acid (b) end-groups are shown. The masses used to obtain the XIC shown are reported in Figure S9. (c, d) Details of the mass spectra extracted between 11.7 and 12.0 min and 12.7 and 13.0 min, respectively. The mass and isotope distribution observed correspond to 5-mer acid-terminated PLA. The times selected correspond to the XIC peak maximum. The MS analysis of acid-terminated PLA with 10, 15, and 20 repeating units is shown in Figure S10.

To assess whether the ESI conditions resulted in in-source fragmentation of PLA and PLGA polymers, we used e-L100-S, mainly consisting of alkyl-terminated PLA (alkyl-PLA), as a model. The range up to roughly 3 or 4 kDa of the SEC-MS data was interpreted, assigning masses based on the ionization agent used and end-group structures. The masses corresponding to acid-terminated structures were monitored using extracted ion chromatograms (XICs) to estimate polymer stability during the SEC-MS process. The total ion chromatogram (TIC) of e-L100-S and XICs of alkyl-terminated PLA and acid-terminated PLA using NaI as an ionization agent are reported in Figure 3.

In the SEC-MS analysis of e-L100-S, a reference alkyl-PLA sample, we observed unexpected masses corresponding to acid-terminated PLA (Figure S11). We, therefore, performed XIC to monitor acid-terminated oligomer masses during the chromatographic process. The SEC separation can partially resolve different oligomers of PLA according to the number of monomeric units. This is, for example, shown by the separation of the alkyl-terminated PLA oligomers (Figure 3a). However, the XIC of acid-terminated species (Figure 3b) revealed the presence of peaks with fronting that extended across a broad SEC range, far exceeding the region where the corresponding alkyl-terminated species were observed. In addition, multimodal elution behavior is observed for the 5 and 10 mer. The analysis of the MS spectra across the different elution regions confirms that masses with similar isotopic patterns and charge states are observed (Figures 3c,d and S12).

To assess the source of the fragmentation, we examined the influence of various ESI instrumental conditions, such as capillary voltage, trap-cone voltage, sampling cone voltage, desolvation temperature, source temperature, and gas pressure. The solvent composition was kept constant at 9:1 ACN/water. The outcomes are summarized in Table S2. Interestingly, the extent to which acid-terminated oligomers were formed varied (between 24% and 5%) depending on the conditions, indicating that fragmentation is likely to occur during ESI. In particular, the sampling cone voltage has a strong influence on end-group analysis results, with a reduction from 24 to 5% of acid-terminated PLA observed when decreasing this voltage from 200 to 100 V (lower voltages do not dimish this further). This indicates that in-source dissociation occurring within the interface between the ESI source and mass spectrometer arises from collisional activation of ions during transfer. Decreasing the cone voltage reduces this activation, thereby limiting the extent of dissociation. However, when the cone voltage is decreased, we observed a significant decrease in the ion intensity, especially for oligomers of higher molecular weight. For example, in Figure S13, we monitor the peak area of PLA 20mer, showing a 2-fold increase in the peak area. We, therefore, concluded that maintaining a voltage of 200 V was useful to extend the molecular weight range of our analysis.

Finally, our results showed that lowering the desolvation gas temperature helped to reduce the fragmentation. However, to maintain efficient ionization with good S/N, we did not reduce this below 150 °C. Other parameters hardly had a significant effect on preventing fragmentation. Experiments using MeOH yielded similar results but with an overall lower ion intensity.

Polyester fragmentation was previously described as occurring via intramolecular transesterification, which generates cyclic polyesters, or through polymer chain scission resulting from a 1,5-hydrogen rearrangement.^23,35^ The proposed mechanism is adapted to PLA/PLGA polyesters and illustrated in Figures S8 (a) and (b). Similarly to what is shown in Figure 3b, the XICs of the alkene-fragments illustrated in Figure S8 displayed peaks between 9 and 13 min (Figure S14). This observation suggests that during the ESI-MS process, both acid-terminated or alkyl-ester-terminated PLA with an alkene as the ω end-group, as well as cyclic PLA, are generated.

In the in-source fragmentation pathway involving 1,5-hydrogen rearrangement, a proton bound to the β carbon in alcohols is needed for the fragmentation to take place. To confirm this we analyzed the polymer PNI, a model aromatic polyester mainly alcohol terminated, which includes neopentyl glycol—a compound that lacks the β proton. (structure reported in Figure 1, values from titration: acid 0.5 alcohol 22.5). In the analysis, the XICs of oligomers with monoacid and diacid end groups did not exhibit clear frontings or multiple peaks during elution (Figure S15). Therefore, we concluded that the fragmentation observed might be related to the higher susceptibility to fragmentation of PLA/PLGA polymers in comparison with polyesters that lacks the β proton.

### 3.3 Alkyl-Terminated End-Group Hydrolysis Can Be Minimized Using Cesium or Rubidium as Ionization Agents

**Figure 4:**
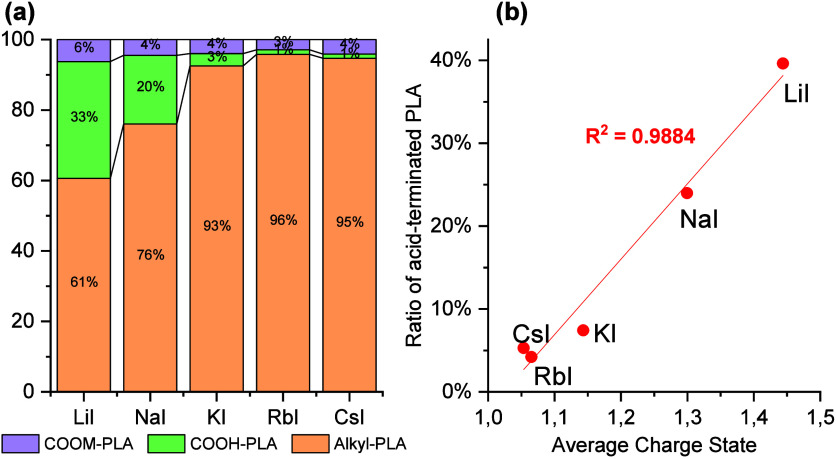
(a) A comparison of the ratios of alkyl-PLA with alkyl and acid end groups using different alkali metal ions in SEC-MS. COOM-PLA, COOH-PLA, and alkyl-PLA stand for alkali metal carboxylated-terminated PLA, acid-terminated PLA, and alkyl-terminated PLA, respectively. The mass spectra were extracted from the range between 11.6 and 13.6 min in SEC-MS. The degree of polymerization characterized in this analysis is up to approximately 60. The ratio was based on the sum of total mass intensities. The average number of the ratio in triplicate analysis was used. The standard deviations of the ratios are shown in Table S3. (b) Correlation between the average charge state and the amount of acid-terminated PLA (including COOM-PLA and COOH-PLA).

Because ESI source parameters proved that the extent of fragmentation could be reduced, we explored the effect of the composition of the makeup flow. We varied the type of alkali-metal ions, keeping their concentration at 0.1 mM, as this is known to be critical in polymer analysis by ESI-MS.^37^ The results of this study are summarized in Figure 4(a). Interestingly, we observed that the intensity of acid-terminated PLA decreased significantly when we used alkali-metal ions with a larger ion radius, such as cesium. For example, a reduction of acidic end-groups from 40% to 5% was observed when LiI was changed to CsI. A detailed analysis of the end group observed using different alkali metal ions is reported in Figure S11.

Our findings are partially supported by what Crescentini et al. described in the context of ion-mobility and tandem-MS analysis of polybutylene adipate oligomers.^38^ Here, alkali metals were shown to affect fragmentation differently in the tandem MS analysis. In particular, Li and Na effectively promoted fragmentation, whereas K, Rb, and Cs did not perform well with respect to Li and Na.

To gain more insight in this phenomenon, we calculated the ACSs for the different metal ions using eq 1.^15,39^ This analysis revealed large differences between the different alkali-metal ions (see Figure 4b). Yin et al. have described that the charge density of polymers, such as PEG, depended on the alkali-metal ions used in ESI-MS, but they did not observe fragmentation.^40^Figures S11 and S12 show results from the analyses of e-L100-S. Interestingly, we found that the intensity of acid-terminated PLA detected in SEC-MS strongly correlated with the ACSs of alkyl-terminated PLA and that the ACS is strongly correlated with the amount of acid-terminated PLA. Comparing, for instance, NaI and CsI as ionization agents, higher ACSs were observed with the former (about 1.3 vs 1.0), resulting in higher acid end-group intensities(24 vs 5%).

The strong correlation between the ACS and the amount of acid-terminated PLA produced by fragmentations suggests that the number of alkali-metal ions added to the polymer chain is proportional to the fragmentation reaction. The ACS is expected to be related to the binding energy between alkali metal ions and the analyte. When using LiI or NaI, the lithium ion or sodium ion binds more strongly to the oxygen atom of the carbonyl group in PLA. This strong binding facilitates charge-remote bond cleavages, such as 1,5-hydrogen rearrangement or intramolecular reactions (as shown in Figure S8), within the polymer backbone without the expulsion of the metal ion. In contrast, when using CsI, the cesium ion suppresses the 1,5-hydrogen rearrangement due to its lower binding energy. Additionally, the correlation between the ACS and the amount of acid-terminated PLA produced through fragmentation can be attributed to the fact that selecting a larger cation increases the mass of the ions generated in the gas phase. This results in a decrease in their center-of-mass energy during activation and transfer, reducing in-source fragmentation.^34,37^

Our observations suggest that the selection of an alkali-metal ion significantly affects the ACSs of PL(G)A, impacting fragmentation in the ESI-MS process. To minimize fragmentation of aliphatic polyesters in the ESI-MS, we suggest that CsI or RbI are the most suitable cation sources for SEC-MS analysis.

**Figure 5:**
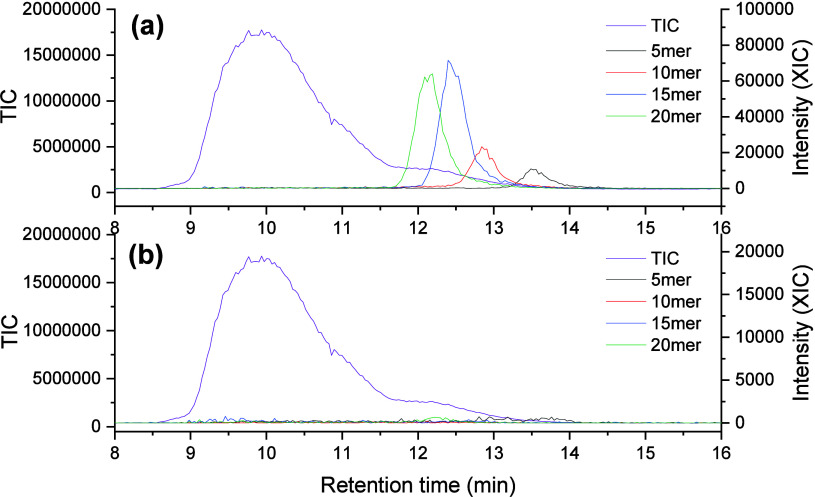
SEC-MS analysis of e-L100-S polymers showing XICs of selected oligomers with alkyl (a) and acid (b) end group using CsI as a cation-providing additive.

Figure 5 shows that the XICs of acid-terminated PLAs showed no discernible signals using CsI. Given the strong dependency of the presence of acid-terminated polymers on the ionization agent, we conclude that fragmentation is the result of in-source processes and not caused by shear degradation during SEC separation.

To further study the impact of ACSs in the PLA hydrolysis, we examined the impact of charge manipulation agents, including supercharging reagents (SCR), such as m-nitrobenzyl alcohol (NBA) and charge-reduction reagents (CRR), such as triethylamine (TEA). These additives have been used to extend the range of detectable molecular weights in ESI-MS analysis.^41−43^ The results are collected in Figures S16, S17, and Table S4. We observed that both additives had only a minor effect on the extent of fragmentation. When using NaI as cation-providing agent, addition of NBA increased the ACS from 1.31 to 1.58 and the amount of acid-terminated PLAs from 24% to 29%. The addition of TEA led to a significant decrease in the ACS (from 1.31 to 1.01), but this did not prevent fragmentations (down from 24% to 20%). The basicity of TEA may also affect the ESI-MS fragmentation.

### 3.4 SEC-MS for the Analysis of PLGA Copolymers

Next we applied the SEC-MS method to the analysis of PLGA copolymers.

**Figure 6:**
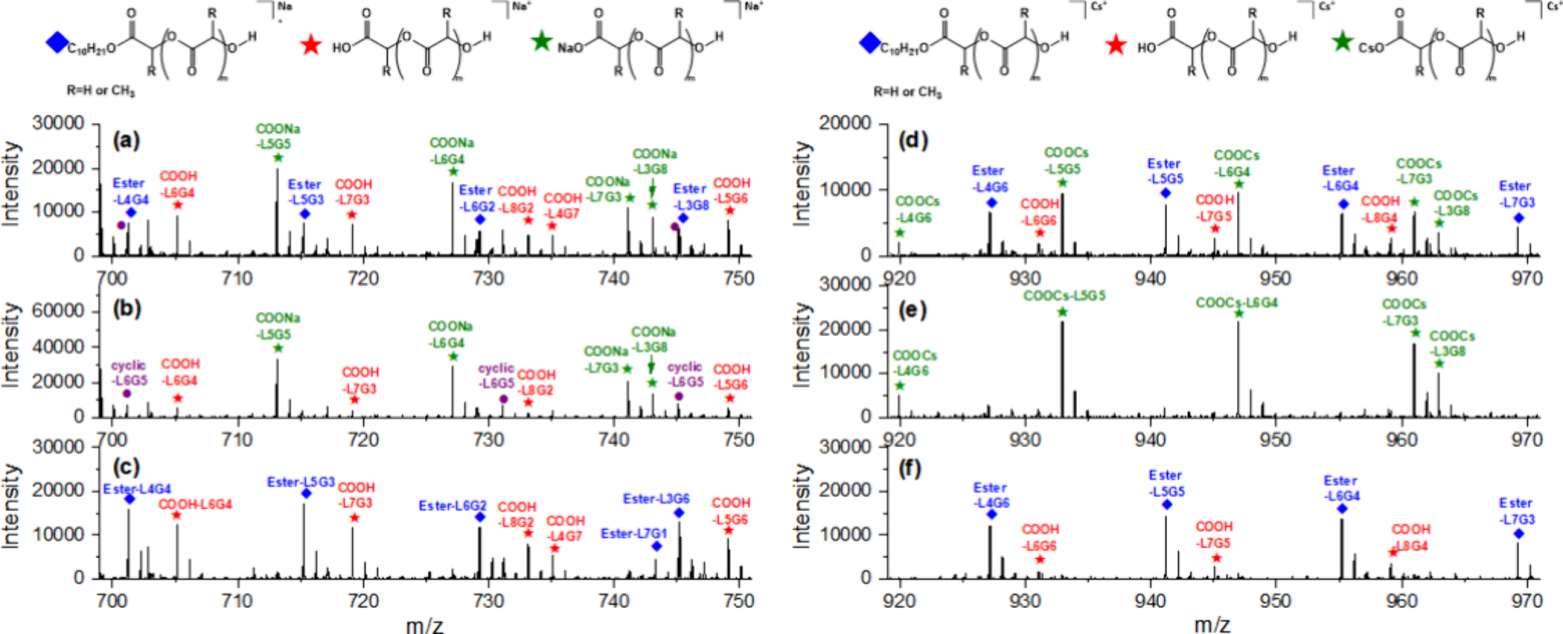
Mass spectra obtained from SEC-MS using NaI or CsI as ionization reagent, extracted between 12.5 and 13.0 min. (a, d) Mass spectra of a mixture of e-L50-S and a-L50-S, at a ratio of 1 to 1 (w/w) with NaI and CsI, respectively. (b, e) Mass spectrum of a-L50-S with NaI and CsI, respectively. (c, f) Mass spectrum of e-L50-S with NaI and CsI, respectively. All peaks displayed in Figure 6 have single charges.

Characterizing both acid and alkyl-terminated PLGA using mass spectrometry is challenging due to the potential presence of isomers with different end groups. Figure S18 provides an example of isomers of PLGAs with acid and alkyl end groups. Since these isomers have the same molecular weight, they cannot be distinguished by their molar masses. SEC-MS analysis using CsI showed a unique feature, i.e., that acid-terminated PLGAs were mainly ionized as alkali-metal carboxylate-terminated PLGAs and rather than as free acids with CsI adducts. This is illustrated in the top part of Figure 6. The significant mass shift (132.9 Da) of Cs adducts facilitated the distinction between acid and alkyl-terminated PLGAs. In comparison, in SEC-MS, with NaI as an ionization agent, acid-terminated PLGA was ionized both as free acid and as alkali-metal carboxylate-terminated.

Figure 6 presents a detail of the mass spectra obtained by SEC-MS analysis using NaI and CsI for a mixture of e-L50-S (alkyl-terminated PLGA) and a-L50-S (acid-terminated PLGA) at a 1:1 weight ratio (a and d) and the mass spectra of a-L50-S and e-L50-S (b-c and e-f), respectively.

Comparing the mass spectra of a-L50-S NaI (Figure 6b) is seen to yield both free acid (around 30% in intensity) and sodium carboxylate, whereas with CsI (Figure 6e), only cesium carboxylate was observed. Consequently, the mass spectrum depicted in Figure 6e is relatively simple compared to that of Figure 6b. We suggest that the prevalence of acid-terminated PLGA in the form of cesium carboxylate when utilizing CsI relates to the higher basicity of Cs ions compared to Na ions, resulting in stronger bonds with carboxylate ions. Thermodynamic property studies of alkali-metal carboxylates in the gas phase in ESI-MS support this hypothesis.^44^

In addition, the analysis of e-L50-S with NaI (Figure 6c) revealed significant relative intensities of acid-terminated PLGA (give 46%). This was not the case for CsI (Figure 6f). To confirm whether the acid-terminated PLGAs included fragment ions, we investigated the XICs using ions detected in Figures 6c and 6f (Figure S19). The XICs of acid-terminated PLGAs using NaI showed broader peaks than those of alkyl-PLGAs, with fronting peaks between 10.0 and 12.0 min, whereas those of acid-PLGAs using CsI showed a similar shape as the alkyl-PLGAs peaks, with no fronting. Based on the discussion of Figure 3 above, this indicated that fragmentation was observed for SEC-MS of PLGA with NaI as an ionization agent, but fragmentation was reduced when using CsI.

### 3.5 Application of SEC-MS Using CsI for the Analysis of Degradation Products of PLGA Copolymers

Finally, we applied the developed SEC-MS method to characterize a PLGA copolymer after chemical degradation. Such studies are crucial to characterize polymer degradability but also to thoroughly understand the microstructures of polyesters, such as sequence distributions or branch structures. Degradation studies are also essential to characterize insoluble polymers.^45,46^ In previous research, Fouquet et al. reported a strategy for characterizing PLGA degradation by coupling SEC offline with MALDI-TOF-MS.^31^ While this method could monitor average molecular weights and the lactic acid to glycolic acid (LA/GA) ratio of collected fractions, it did not address the characterization of acid-terminated PLGA, one of the main degradants in PLGA biodegradation. An example of the characterization of PLGA degradants, which focused on acid-terminated PLGA, was reported by Li et al.^47^ However, the report did not address the simultaneous characterization of PLGAs with different end groups, including acid and alkyl-terminated PLGAs.

**Figure 7:**
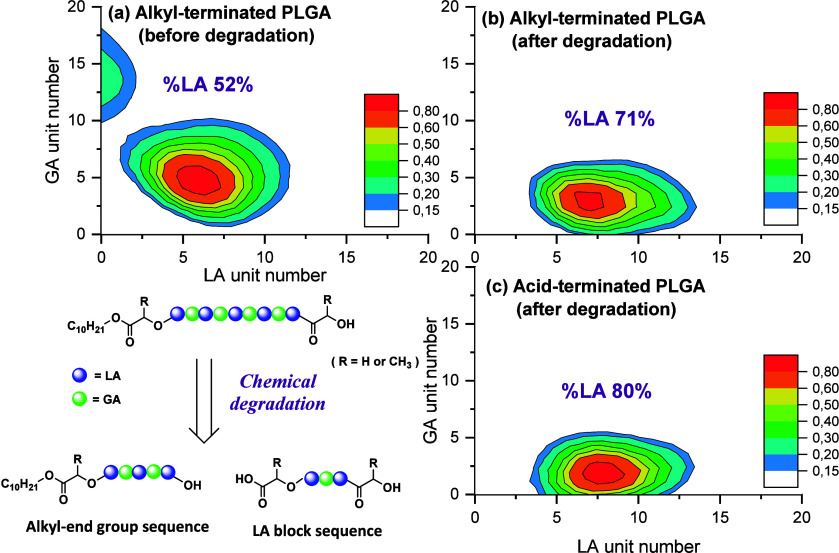
Chemical composition plots for e-L50-S in the 12.5–13.0 min fraction extracted from SEC-MS. (a) Alkyl-terminated PLGA in e-L50-S before chemical degradation, (b) alkyl-terminated PLGA in e-L50-S after chemical degradation, and (c) acid-terminated PLGA in e-L50-S before chemical degradation. The heat maps were made based on the peak characterization using MSPolyCalc. The mass spectra and peak lists are provided in Figure S21, Tables S6 and S7, respectively. The colors in the heat maps indicate the relative intensities, with the highest assigned peak set at unity.

Our method was applied to study the chemical degradation of e-L50-S (LA/GA = 50/50, α and ω end-groups are C_10_H_21_ and OH.). The SEC-UV analysis, shown in Figure S20, traced the chemical degradation, revealing an apparent reduction in the molecular weight of e-L50-S and the formation of PLGA oligomers (Mn and Mw are shown in Table S5). Figure 7 illustrates the results of the characterization of ester and acid-terminated PLGA oligomers before and after the degradation of e-L50-S.

The SEC-MS analysis, derived from the MS intensity of the species of roughly 0.5 to 3 kDa, revealed that, before degradation, the polymer contained only alkyl end groups and 52% LA. These results are in agreement with NMR results (also 52% LA). Figure 7a reveals that a homogeneous chemical composition in LA and GA repeating units is present, with the polymer being formed mostly from equal numbers of GA and LA units. As a result of the chemical degradation, acid-terminated polymers are formed, with 45% of the end group detected being acids (Table S8). It is important to note that these values should not be considered quantitative as the accuracy of the ratio of acid-terminated oligomers obtained through SEC-MS has not been established. End-group analysis by ^1^H NMR was attempted but was not successful due to the complex spectra resulting from the presence of oligomers of varying lengths (Figure S22).

The percentage of LA in the observed acid-terminated polymer is 80%, with most of the polymers being rich in LA with lengths between 4 and 13 and including, on average, approximately 2 GA units. This suggests that the process of hydrolysis preferentially leads to the hydrolysis of GA units, and because of this degradation, rich-LA oligomers can be observed. A rate of hydrolysis of bonds GA-GA > GA-LA or LA-GA > LA-LA is also in accordance with chemical-degradation studies performed under milder conditions by Li et al.^48,49^ The order may be explained by the greater hydrophilicity of the GG bonds and increased steric hindrance of methyl groups in L-containing diads.^48,49^ Although these results are only preliminary, we suggest that a similar analytical approach may potentially be used to investigate the blockiness of PLGA copolymers.

## 4 Conclusion

This study aimed to evaluate whether in-source fragmentation of the biodegradable polymer PLGA occurs during SEC-MS analysis and to establish analytical conditions at which fragmentation is suppressed.

Our results show that the stability of polyesters during SEC-MS analysis is strongly influenced by the choice of alkali metal salt used for ionization. Using LiI or NaI to ionize PLA aliphatic polyesters was shown to lead to fragmentation during ESI-MS, while fragmentation was suppressed when using CsI. Similar results were also obtained for PLGA, with mainly cesium-carboxylated end-groups being observed when characterizing acid-terminated polymers. This simplified the assignment of polymer peaks in the mass spectra, removing instances in which isomeric PLGA structures could not be distinguished. Our method allowed determining both end-groups and chemical composition of the polymers. Finally, when used in combination with chemical degradation, the described method showed the potential to yield insight in polymer blockiness, particularly for alkyl-end-capped PLGA. The proposed method may provide deeper insights on long-chain polymers, which cannot be obtained by NMR analysis. Because the elucidation of the (local) degree of randomness is an important topic in the field of drug delivery, the SEC-MS method is expected to contribute to further developments in sequence-controlled PLGA.^48,50−53^

## Supporting Information Available

(i) NMR, NPLC, and SEC-UV analysis of starting polymers, (ii) SEC-MS method parameters, (iii) in-source fragmentation analysis and effects of different ionization agents, and (iv) SEC and NMR results from the chemical degradation of PLGA (PDF)
