# Supplementary material for: Size-Exclusion Chromatography–Electrospray-Ionization Mass Spectrometry To Characterize End Group and Chemical Distribution of Poly(lactide-co-glycolide) Copolymers
Source: J Am Soc Mass Spectrom. 2025 Mar 31;36(5):980–90. doi: 10.1021/jasms.4c00447 (PMC12063183; doi:10.1021/jasms.4c00447)
Supplement: Supplementary file 1 — js4c00447_si_001.pdf [file js4c00447_si_001.pdf]

Size-exclusion chromatography–electrospray-ionization mass spectrometry to characterize end group and chemical distribution of poly(lactide-co-glycolide) co-polymers

Supporting Information

Masashi Serizawa [a,b,c*] , Pieter van Delft [d], Peter J. Schoenmakers [a,b], Ron A. H. Peters [a,b,e], Andrea F.G. Gargano [a,b**]

[a.] Van't Hoff Institute for Molecular Sciences, University of Amsterdam, Science Park 904, 1098 XH Amsterdam, The Netherlands
[b] Centre for Analytical Sciences Amsterdam, Science Park 904, 1098 XH Amsterdam, The Netherlands
[c] Material Characterization laboratory, Mitsubishi Chemical Corporation, 1000 Kamoshida-cho, Aoba-ku, Yokohama-shi, Kanagawa 227-8502, Japan
[d] Corbion, 4200 AA Gorinchem, The Netherlands
[e] Covestro, TAP, Group Innovation and Sustainibility, Sluisweg 12, Waalwijk, 5145 PE, the Netherlands

* Corresponding author
m.serizawa@uva.nl
** Corresponding author
a.gargano@uva.nl

Table of Contents

NMR analysis of starting polymers

Figure S1. ^{1}H-NMR spectra of e-L100-S S3

Figure S2. ^{1}H-NMR spectra of e-L50-S S4

Figure S3. ^{1}H-NMR spectra of a-L50-S S5

Figure S4. ^{1}H-NMR spectra of PNI S6

NPLC analysis of aliphatic polyesters

Figure S5. NPLC chromatograms of aliphatic polyesters S7

SEC-UV analysis of starting polymers

Figure S6. SEC-UV chromatograms (UV 220 nm) of aliphatic polyesters S8

Figure S7. SEC-UV chromatograms (UV 254 nm) of PNI S9

SEC-MS method parameters

Table S1. Comparison of the analytical greenness S10

Table S2. Average charge state (ACS) of e-L100-S and the number of the ratio of acid-terminated PLA, employed using SEC-MS with different instrumental conditions S11

In-source fragmentation analysis and effects of different ionization agents

Figure S8. In-source fragmentation mechanisms S12

Figure S9. Mass spectra of e-L100-S S13

Figure S10. Expanded mass spectra of e-L100-S S14

Figure S11. Abundances of polylactic acid in MS spectra comparing different salts..S**Error! Bookmark not defined.**

Figure S12. Abundances of polylactic acid with different charge states in MS spectra .... S**Error! Bookmark not defined.**

Figure S13. Correlation between the sampling cone voltage used in SEC-MS and the XIC area intensities of alkyl-ester-terminated PLA S17

Figure S14. SEC-MS analysis of e-L100-S showing XICs of fragment ions using NaI or CsI as a cation additive S17

Figure S15. SEC-MS analysis of PNI polymers showing XICs S19

Figure S16. Abundances of polylactic acid in MS spectra using a supercharging agent S20

Figure S17. Abundances of polylactic acid in MS spectra employing SEC-MS using TEA S21

Table S3. A number of the ratios of alkyl and acid-terminated PLA .....S2**Error! Bookmark not defined.**

Table S4. ACS of e-L100-S and the number of the ratio of acid-terminated PLA, employed using charge manipulations S22

Results from chemical degradation of PLGA

Figure S18. An example of isomers between alkyl-terminated and acid-terminated PLGA S23

Figure S19. XICs of e-L50-S with alkyl (a) and acid (b) end group S24

Figure S20. SEC-UV chromatograms of e-L50-S before and after degradation S25

Figure S21. MS spectra of e-L50-S before and after degradation S26

Figure S22. ^{1}H-NMR spectra of e-L50-S before and after chemical degradation S27

Table S5. Polystyrene equivalent molar masses and polydispersity of degraded PLGA S28

Table S6. Characterization of product ions detected in MS spectra of e-L50-S before chemical degradation S29

Table S7. Characterization of product ions detected in MS spectra of e-L50-S after chemical degradation S32

Table S8. PLGA contents with different end groups S35

NMR analysis of starting polymers

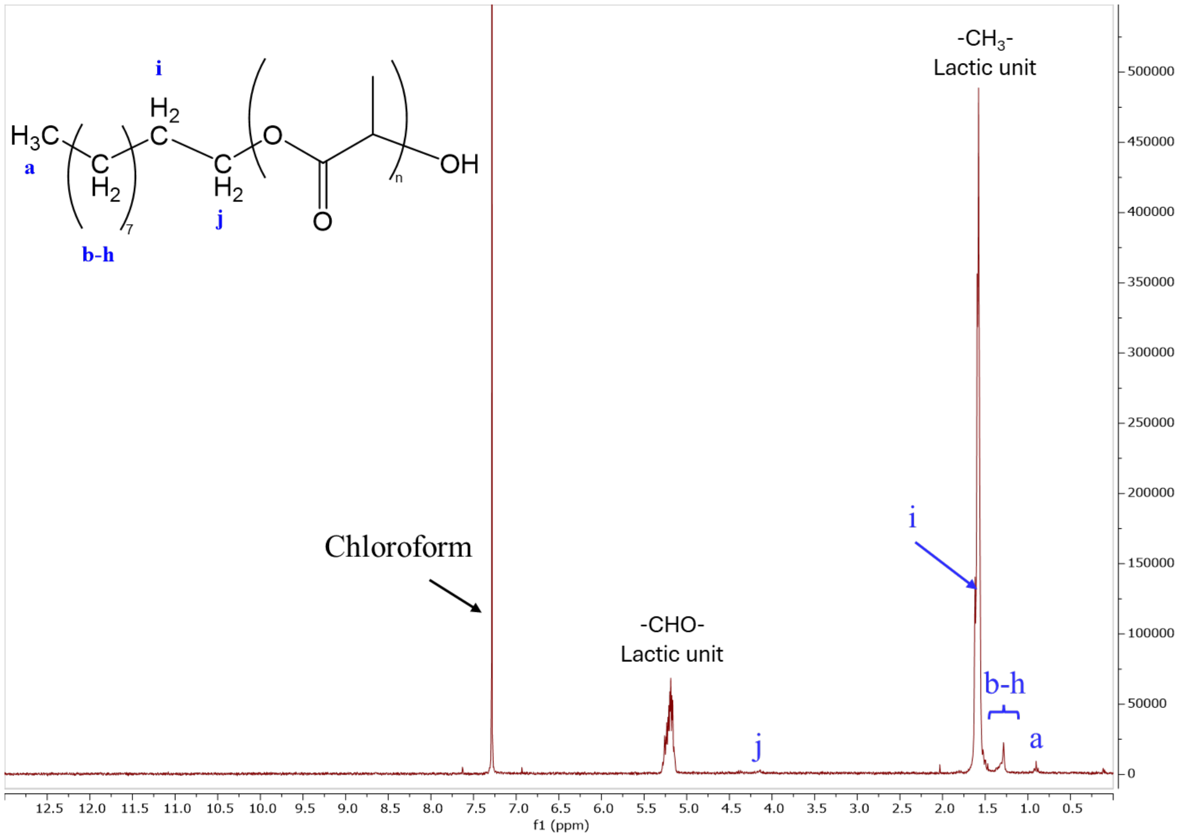

Figure S1. ^{1}H-NMR (300 MHz, Chloroform-*D₁*) spectra of e-L100-S

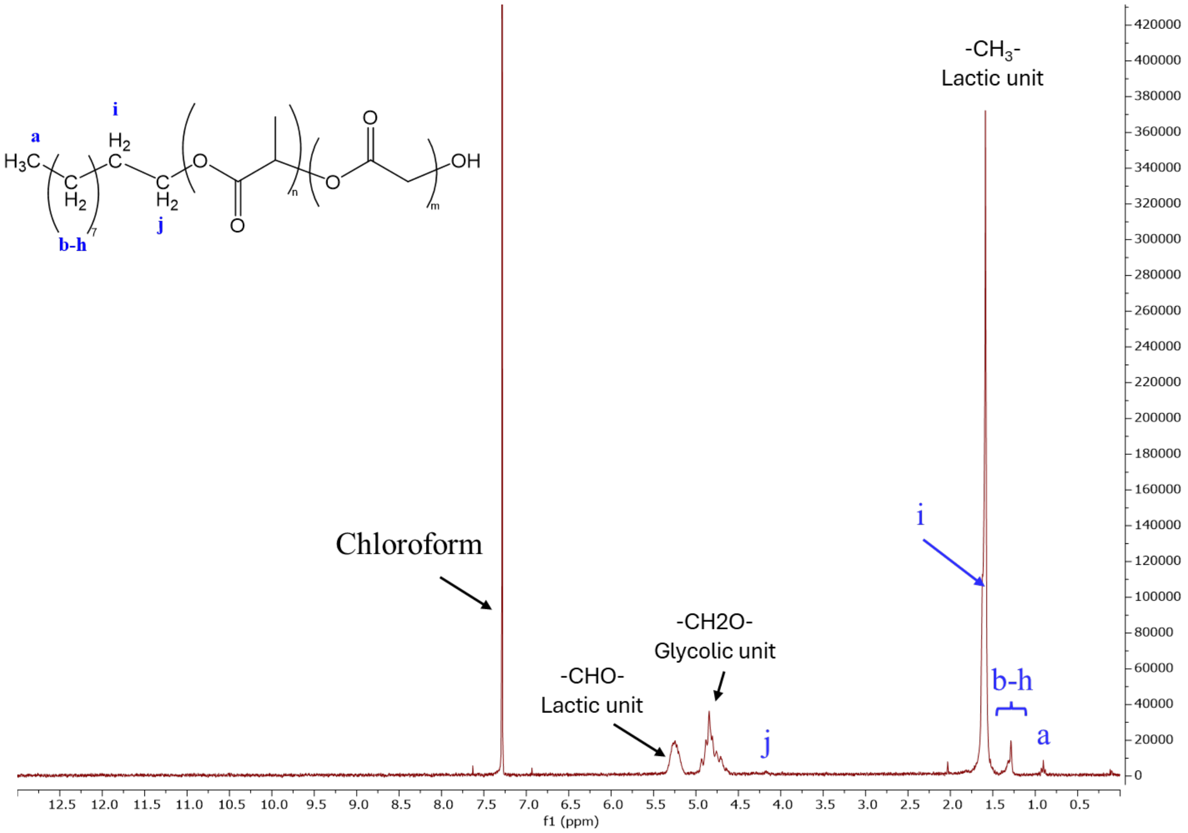

Figure S2. ^{1}H-NMR (300 MHz, Chloroform-D_1) spectra of e-L50-S

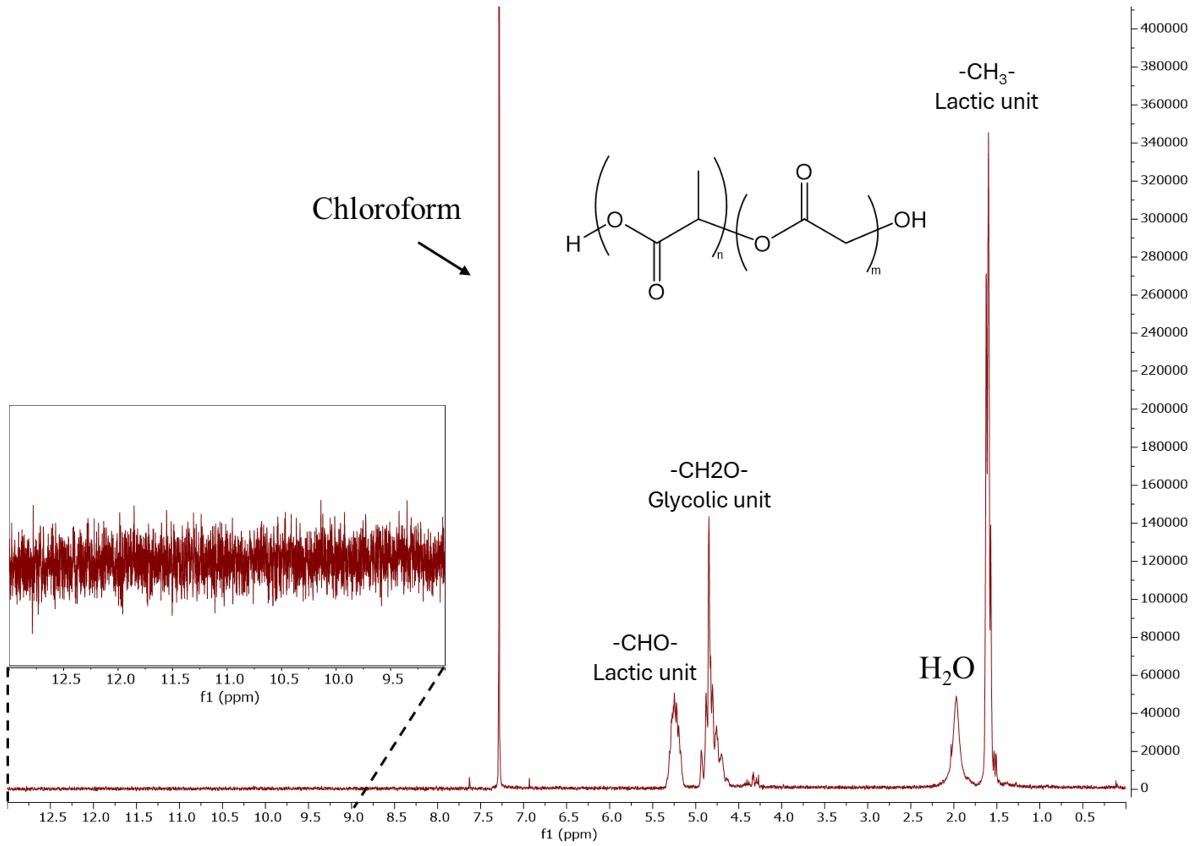

Figure S3. ^{1}H-NMR (300 MHz, Chloroform-D1) spectra of a-L50-S

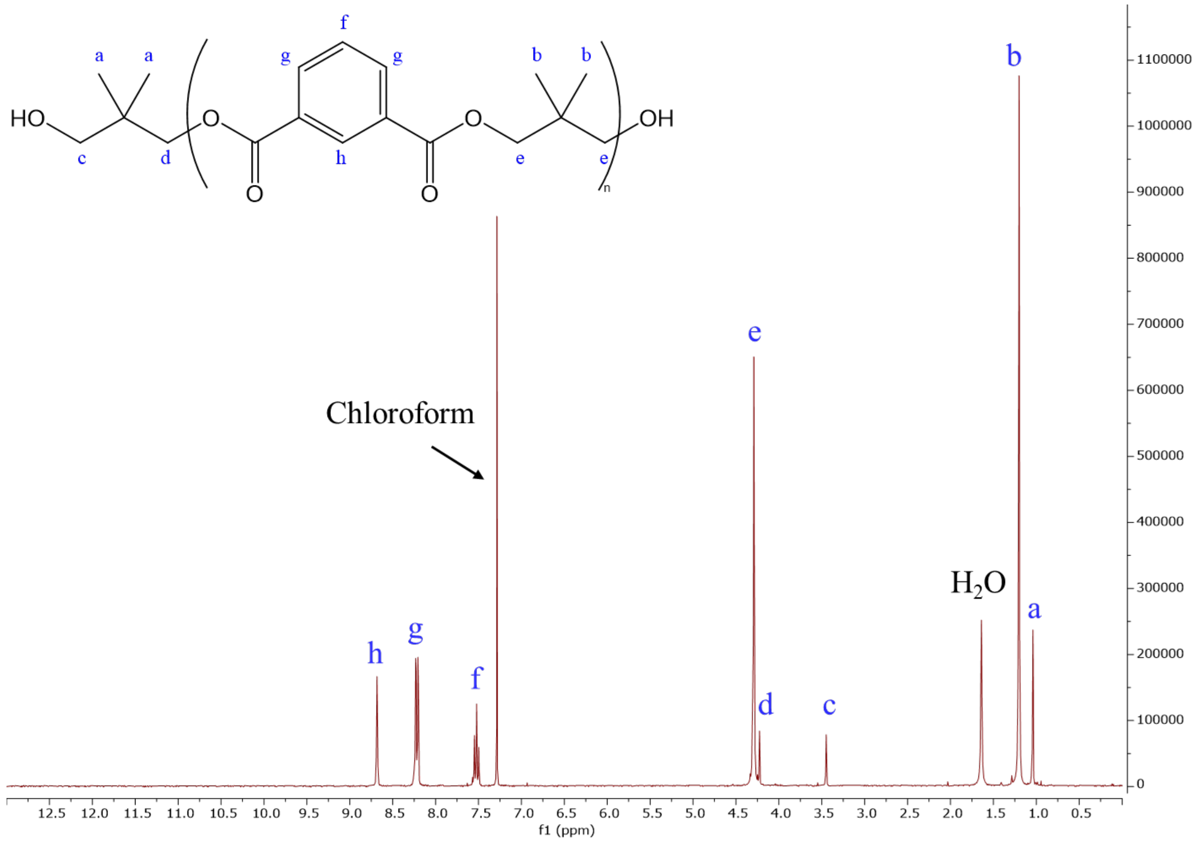

Figure S4. ^{1}H-NMR (300 MHz, Chloroform-D_1) spectra of PNI

NPLC analysis of aliphatic polyesters

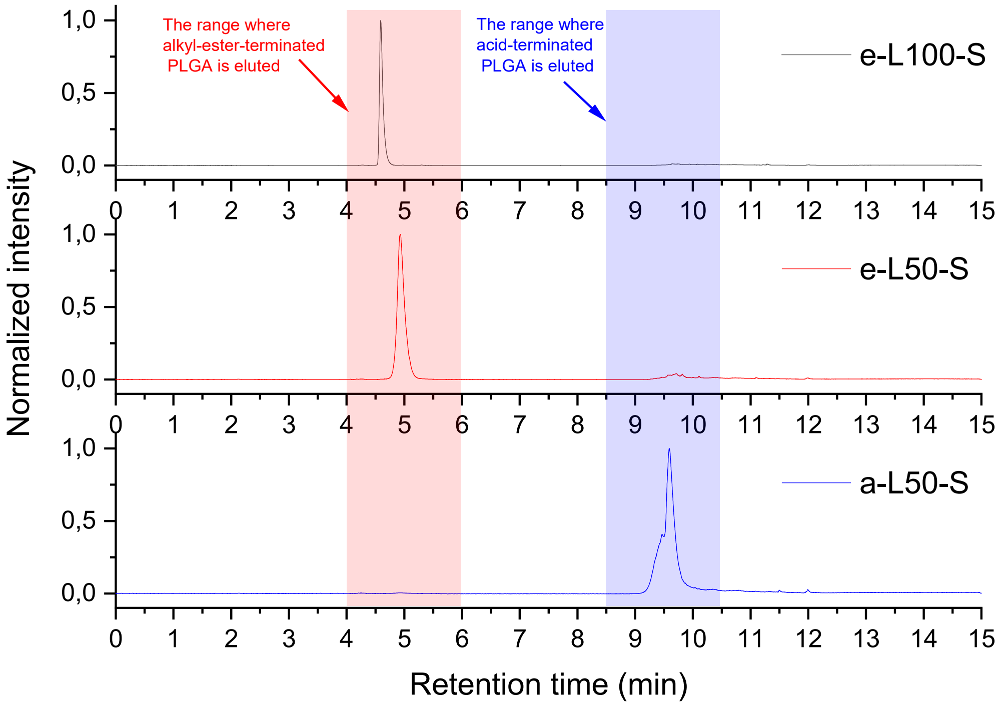

Figure S5. NPLC chromatograms of aliphatic polyesters

SEC-UV analysis of starting polymers

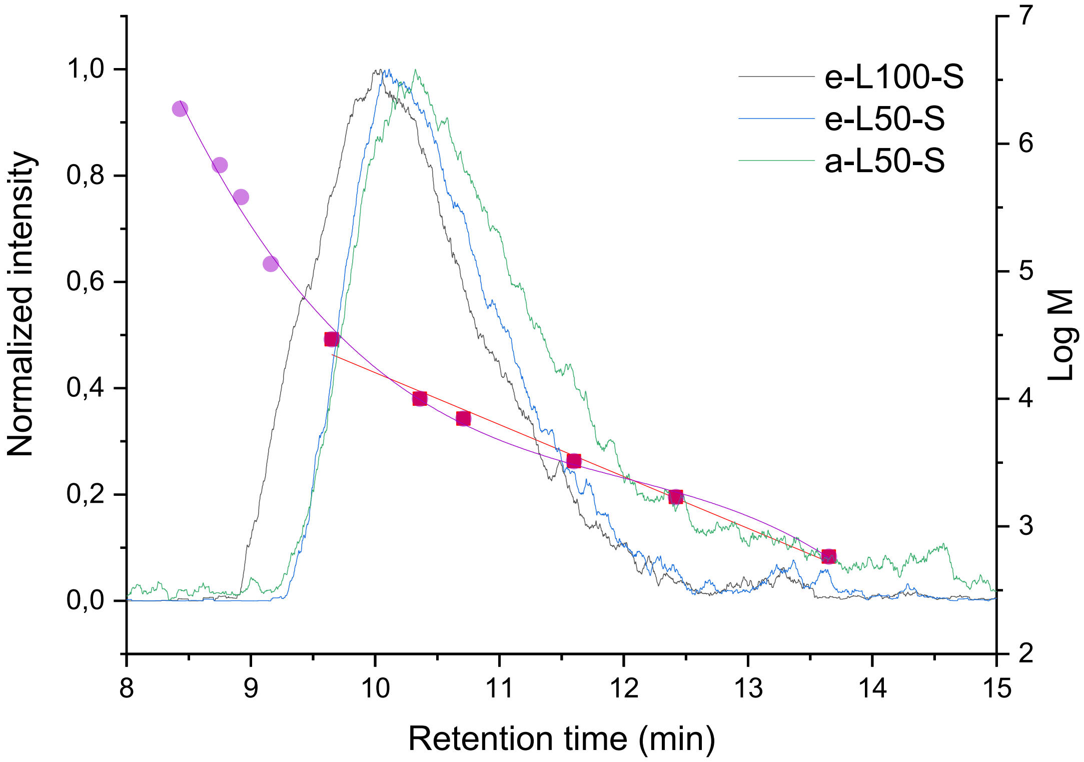

Figure S6**.** SEC-UV chromatograms (UV 220 nm) of aliphatic polyesters in Table 1
Molecular weight parameters indicated in Table 1 are determined based on a PS-based calibration. The M_n and M_w values were calculated using the following equations where S is the signal and M the mass:

$$\overline{M_n} = \frac{\sum(S_t * M_t)}{\sum S_t} \quad \text{(S1)}$$

$$\overline{M_w} = \frac{\sum(S_t * M_t) * M_t}{\sum(S_t * M_t)} \quad \text{(S2)}$$

$$PDI = \frac{M_w}{M_n} \quad \text{(S3)}$$

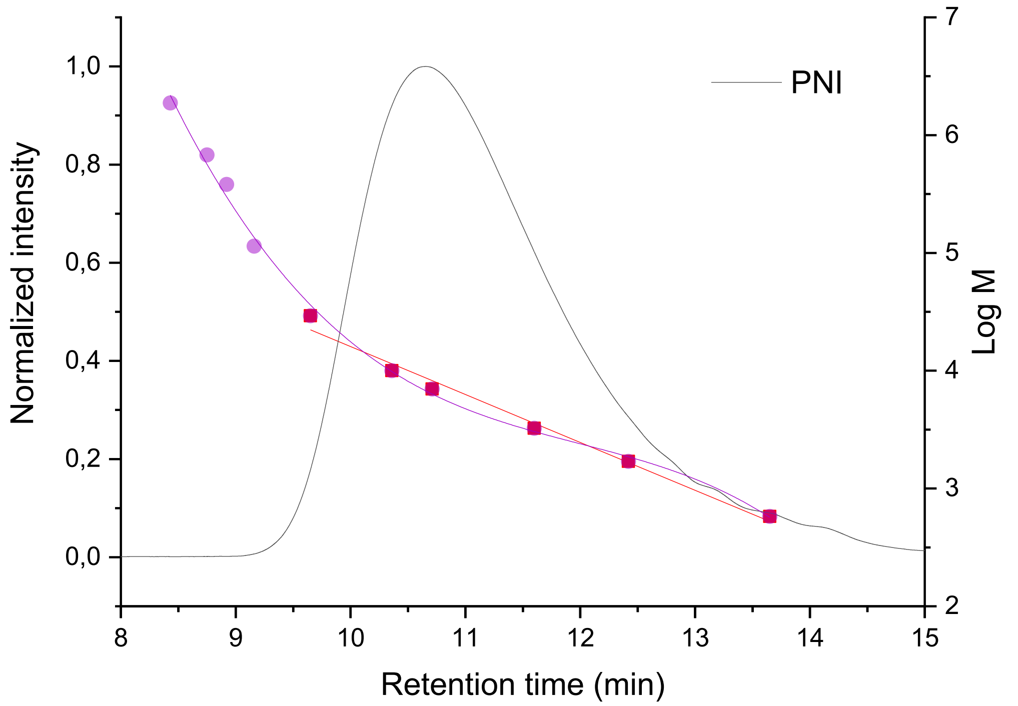

Figure S7. SEC-UV chromatograms (UV 254 nm) of PNI (an aromatic polyester) in Table 1

SEC-MS method parameters

Table S1. Comparison of the analytical greenness between analytical flow and microflow method. Data obtained comparing the method described with an equivalent method run at the same linear flow velocity using a 4.6 mm ID column.

Method	Analytical flow	Microflow
Greenness Score	270.74	143.01
Instrument Energy Score	63.54	63.54
Solvent Energy Score	198.16	75.38
Solvent EHS Score	9.04	4.10
Number of analytes of interest	5	5
Number of injections/runs for one full analysis	10	10
Flow rate (mL/min)	**0.20**	**0.04**
Run time (min/injection)	18.5	18.5
Gradient (Time 0 min)	**91%A, 9%B**	**67%A, 33%B**
Gradient (Time 18.5 min)	**91%A, 9%B**	**67%A, 33%B**
Mobile Phases (Mobile phase A)	THF (100%)	THF (100%)
Mobile Phases (Mobile phase B)	Acetonitrile (90%) /Water (10%)	Acetonitrile (90%) /Water (10%)
Sample prep volume (mL)	1	1
Number of Sample preps	10	10
Solvent	THF (100%)	THF (100%)

Table S2. Average charge state (ACS) of e-L100-S and the number of the ratio of acid-terminated PLA, employed using SEC-MS with different instrumental conditions*

Sample cone voltage (V)	Capillary voltage (eV)	trap collision energy (eV)	Desolvation temperature (°C)	Gas pressure (L/h)	ACS Average	N_{acid} Average
200	3.5	10	350	850	1.30	24%
200	2.5	10	350	850	1.42	16%
200	1.5	10	350	850	1.49	18%
200	3.5	5	350	850	1.40	16%
200	3.5	20	350	850	1.41	18%
200	3.5	10	150	850	1.42	14%
200	3.5	10	350	400	1.42	17%
150	3.5	10	350	850	1.39	8%
100	3.5	10	350	850	1.38	5%
50	3.5	10	350	850	1.34	5%

*Initial experiments were performed to establish the source temperature effect, testing temperatures between 100-150 °C. Limited differences were observed; therefore, a reference temperature of 120 °C was selected.

In-source fragmentation analysis and effects of different ionization agents

(a) In-source fragmentation generating alkene-terminated PLGA in gas phase

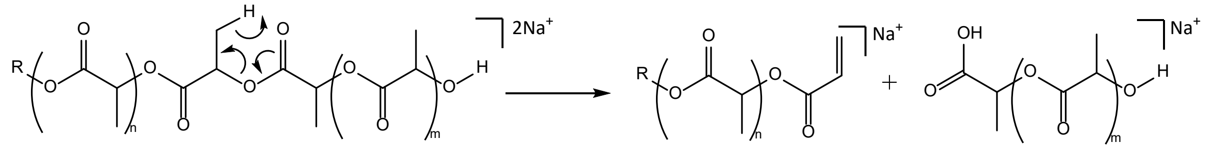

(b) In-source fragmentation generating cyclic PLGA in gas phase

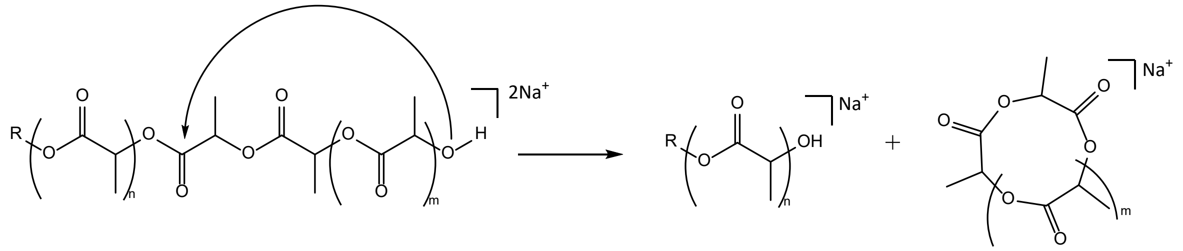

Figure S8. (a) In-source fragmentation mechanisms in which acid-terminated PLA is produced via 1,5-H rearrangement. R is proton or $C_{10}H_{21}$. (b) In-source fragmentation mechanisms in which cyclic PLA is produced via intramolecular fragmentation.

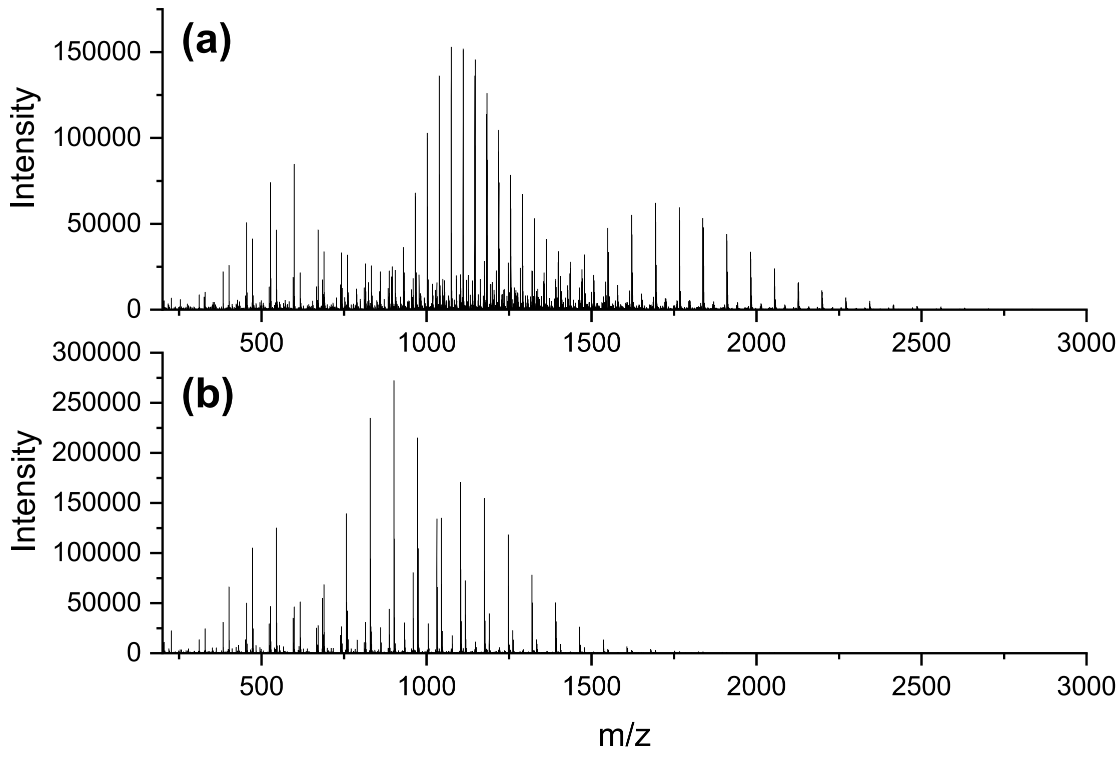

Figure S9. (a) Mass spectra of e-L100-S extracted between 11.7 and 12.0 minutes of SEC-MS analysis, (b) Mass spectra extracted between 12.7 and 13.0 minutes

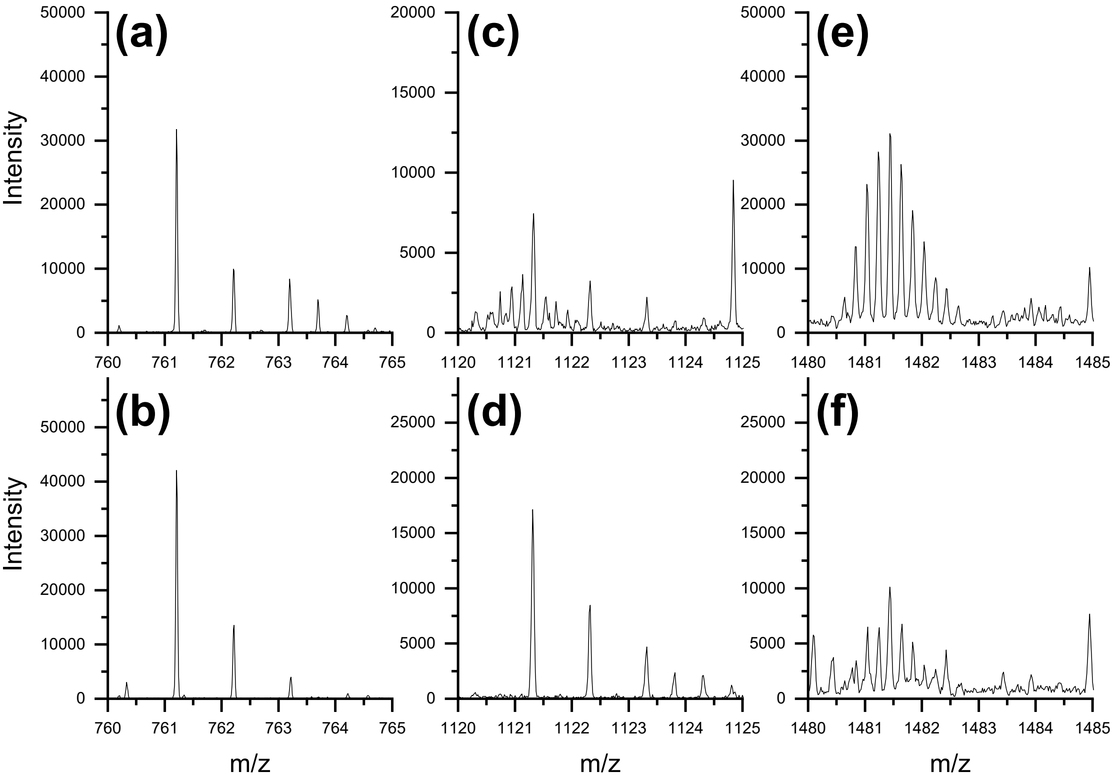

Figure S10. (a) Expanded mass spectra of e-L100-S extracted between 11.7 and 12.0 minutes of SEC-MS analysis, where a fronting peak was detected in the XIC of 10mer acid-terminated PLA. (b) Expanded mass spectra extracted between 12.7 and 13.0 minutes, where a main peak was detected in the XIC of 10mer acid-terminated PLA. (c) Expanded mass spectra extracted between 10.7 and 11.0 minutes, where a fronting peak was detected in the XIC of 15mer acid-terminated PLA. (d) Expanded mass spectra extracted between 11.7 and 12.0 minutes, where a main peak was detected in the XIC of 15mer acid-terminated PLA. (e) Expanded mass spectra extracted between 10.2 and 10.5 minutes, where a fronting peak was detected in the XIC of 20mer acid-terminated PLA. (f) Expanded mass spectra extracted between 10.7 and 11.0 minutes, where a main peak was detected in the XIC of 20mer acid-terminated PLA.

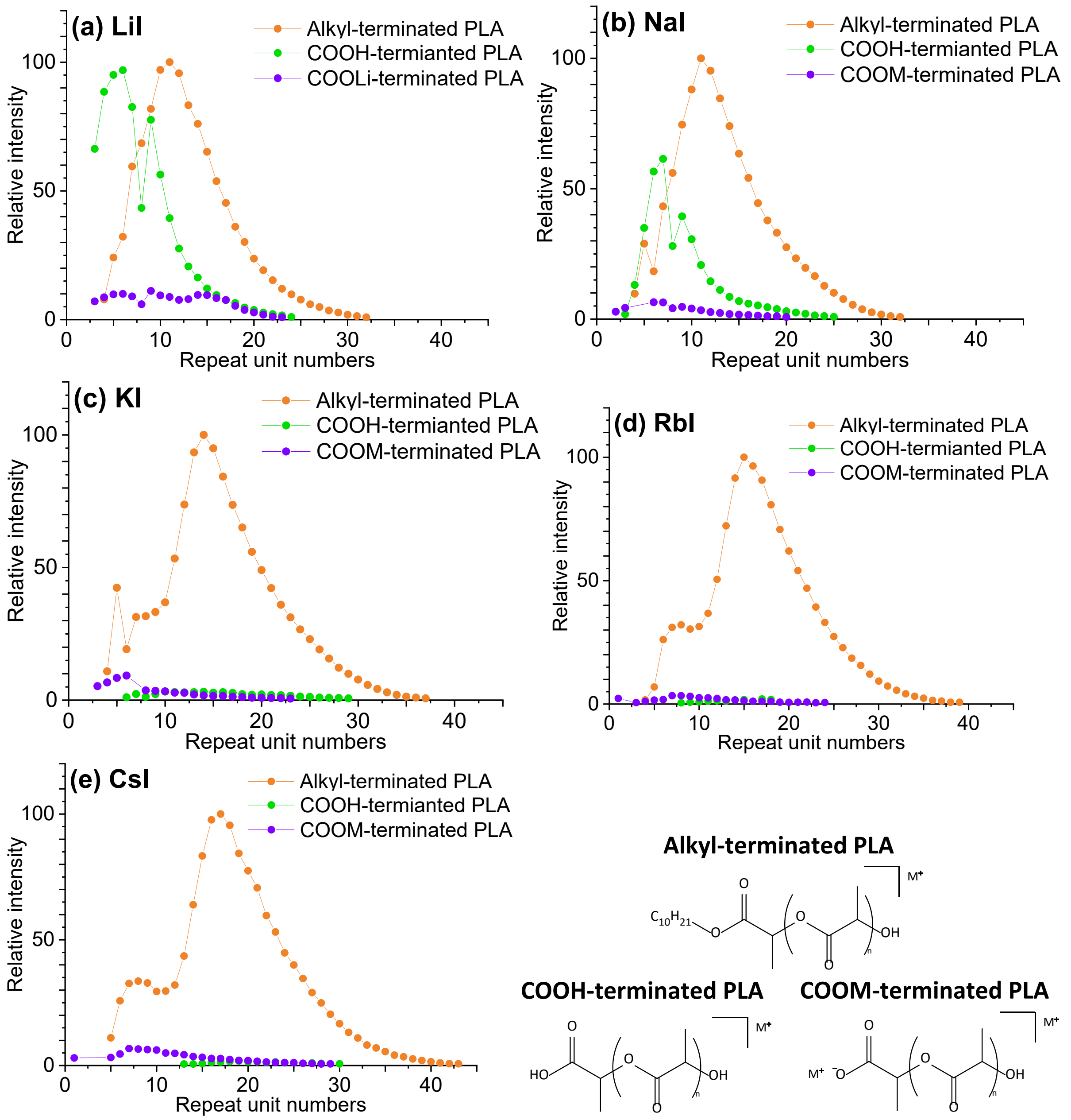

Figure S11. Abundances of polylactic acid end groups in MS spectra collected by SEC-ESI-MS were compared to the use of different salts in makeup B flow. (a) 10% v/v 1mM LiI aq in acetonitrile, (b) 10% v/v 1mM NaI aq in acetonitrile, (c) 10% v/v 1mM KI aq in acetonitrile, (d) 10% v/v 1mM RbI aq in acetonitrile, (e) 10% v/v 1mM LiI aq in acetonitrile.

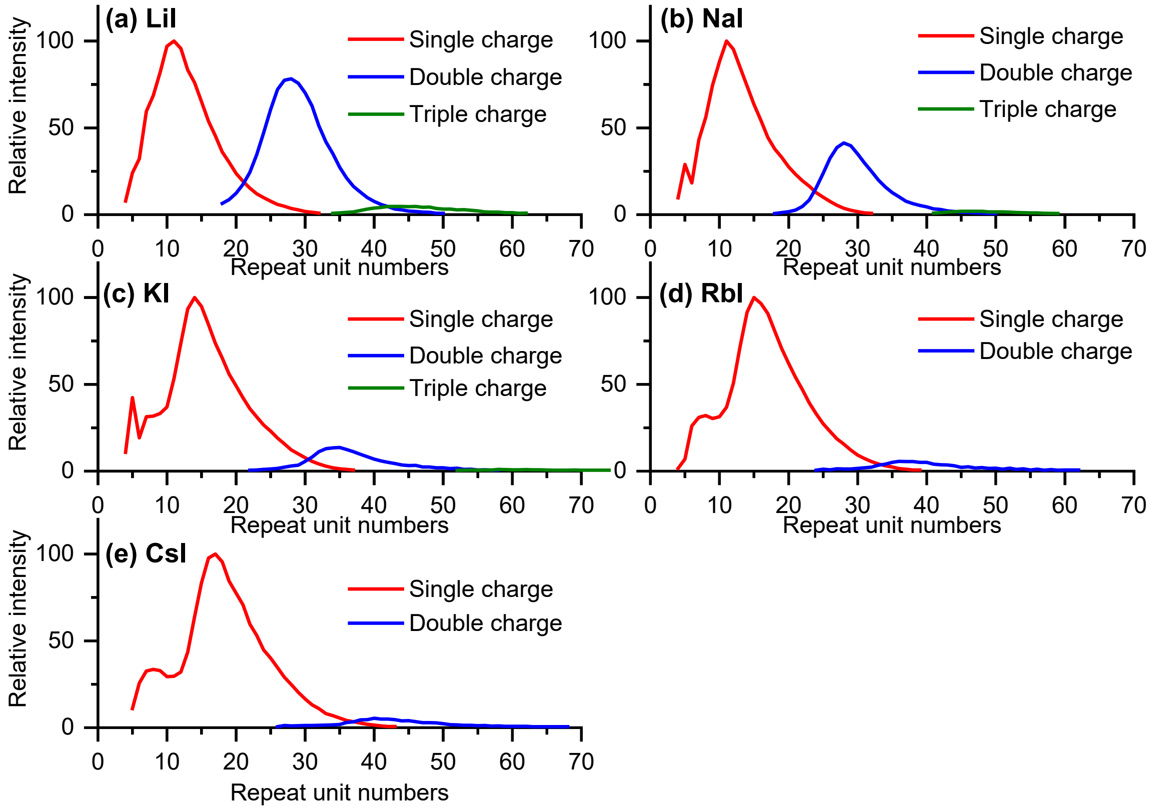

Figure S12. Abundances of e-PLA with different charge states in MS spectra collected by SEC-ESI-MS were compared to the difference in alkali ions. (a) LiI (b) NaI (c) KI (d) RbI (e) CsI The average charge states of the MS spectra depicted in (a), (b), (c), (d), and (e) were 1.44, 1.30, 1.14, 1.07 and 1.05, respectively.

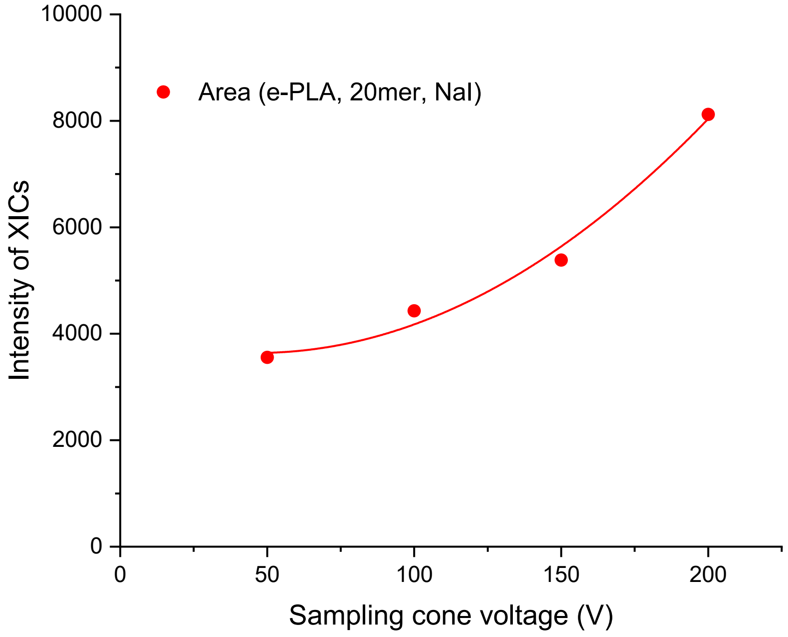

Figure S13. Correlation between the sampling cone voltage used in SEC-MS and the XIC area intensities of alkyl-ester-terminated PLA with 20mer

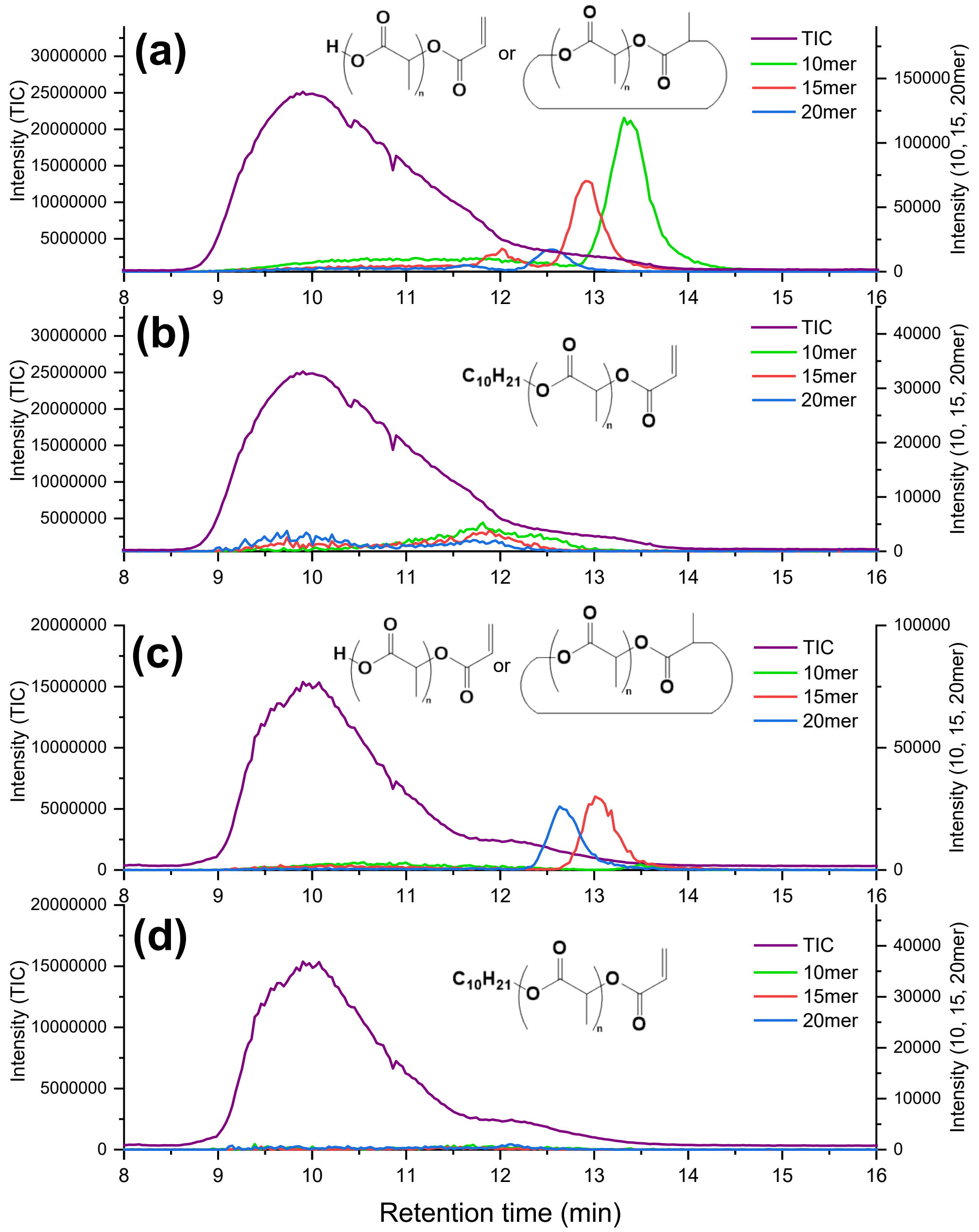

Figure S14. SEC-MS analysis of e-L100-S, showing XICs of fragment ions using NaI or CsI as a cation additive. TIC and XICs of PLA oligomers (10-20 repeating units) that have α and ω end-groups H and $C_3H_3O_2$ with NaI and CsI, respectively; (a and c) TIC and XICs of PLA oligomers (10-20 repeating units) that have α and ω end-groups $C_{10}H_{21}$ and $C_3H_3O_2$ with NaI and CsI, respectively; (b and d). It is noted that XICs of PLA with α and ω end-groups H and $C_3H_3O_2$ include cyclic PLA ions due to the same molar mass.

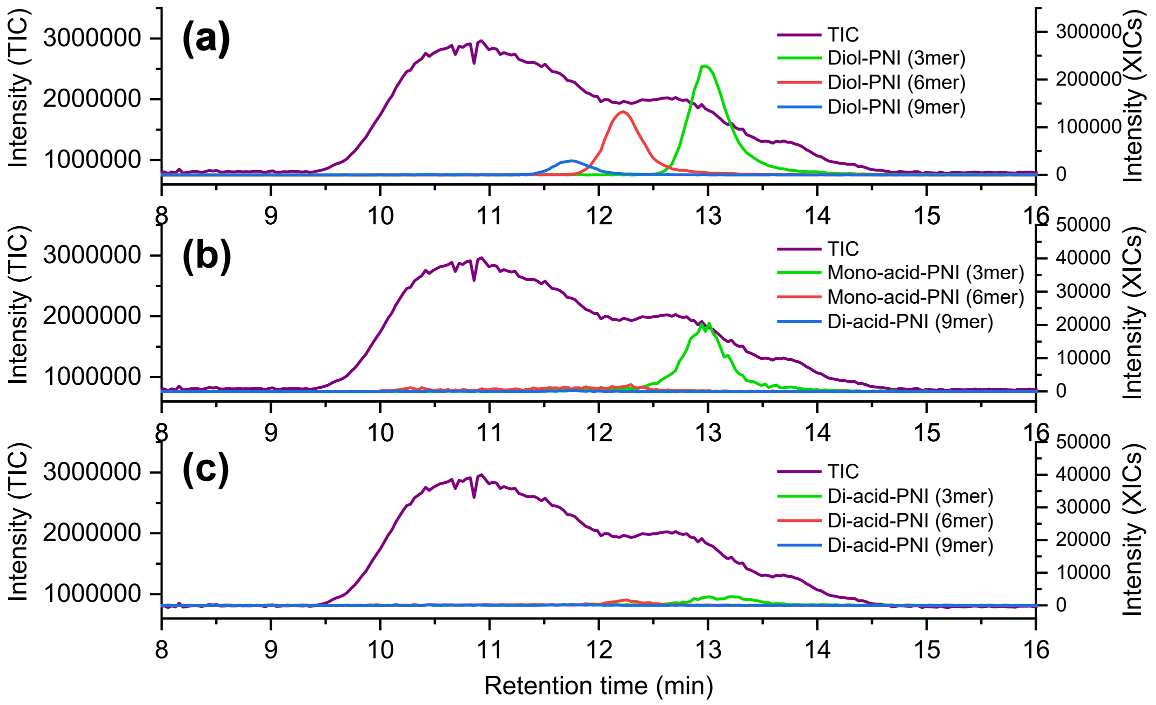

Figure S15. SEC-MS analysis of PNI polymers, an aromatic polyester with an acid value of 3.3 and an OH value of 41.6, showing XICs of selected oligomers with diol (a), mono-acid (b), and di-acid (c) end group using NaI as a cation additive.

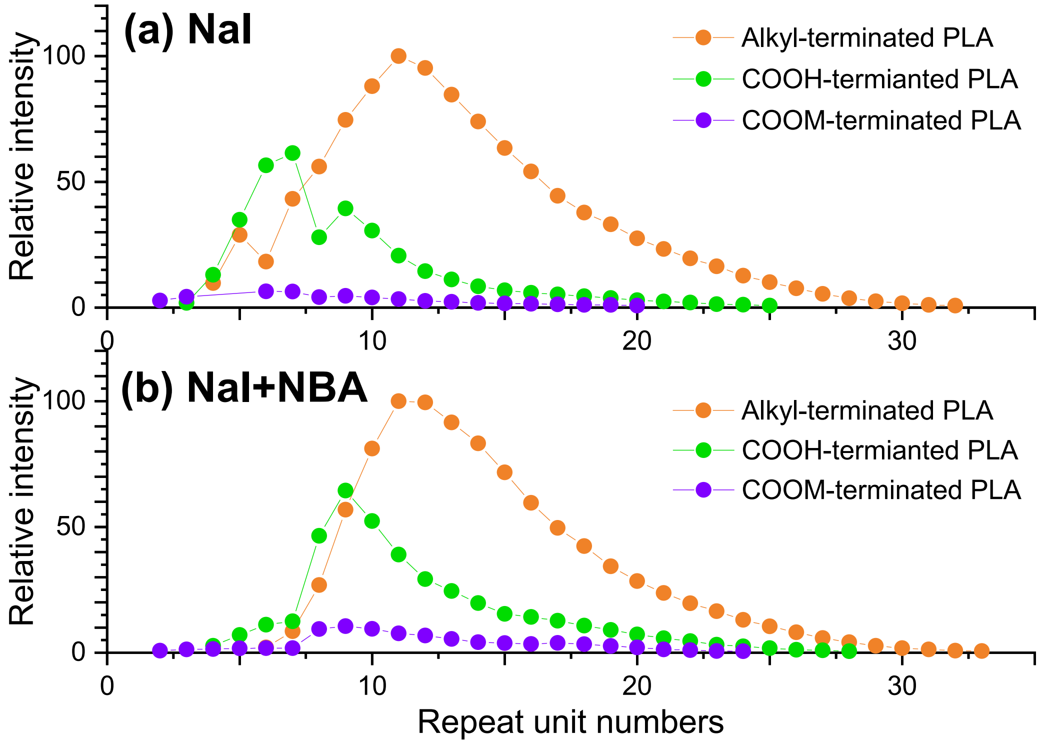

Figure S16. Abundances of polylactic acid in MS spectra using a supercharging agent. (a) 10% v/v 1mM NaI aq in acetonitrile, (b) 0.5% v/v NBA (3-nitrobenzyl alcohol) with 10% v/v 1mM NaI aq in acetonitrile

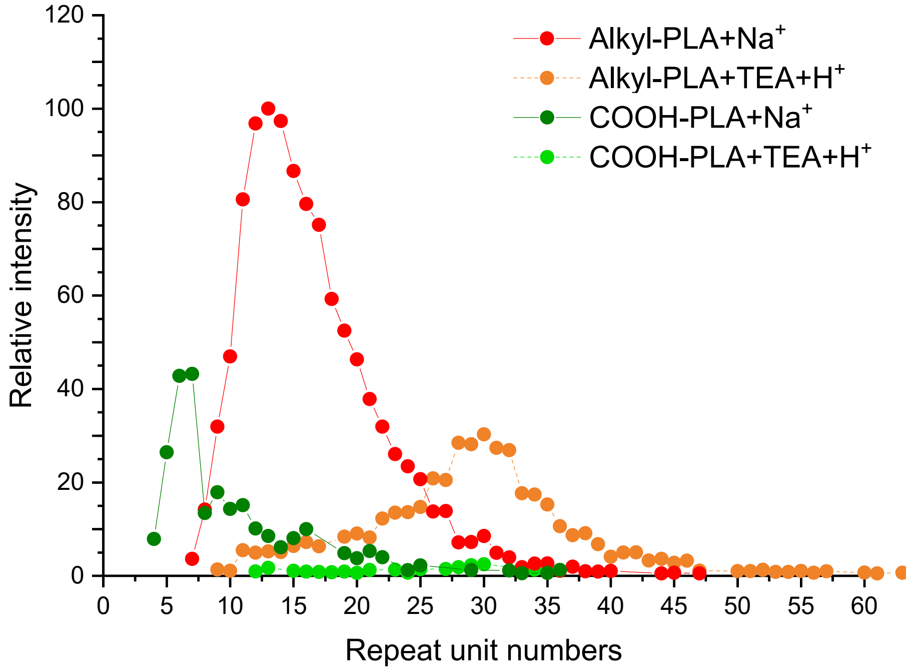

Figure S17. Abundances of polylactic acid in MS spectra collected by SEC-ESI-MS using TEA as a charge reduction reagent 10% v/v 12 mM TEA aq in acetonitrile

Table S3. umber of the ratio of alkyl and acid-terminated PLA

	LiI Mean±SD	NaI Mean±SD	KI Mean±SD	RbI Mean±SD	CsI Mean±SD
Alkyl-terminated PLA	60.4±2.0%	76.0±0.6%	92.6±0.3%	95.8±0.2%	94.7±0.8%
COOH-terminated PLA	33.4±1.6%	19.5±0.4%	3.5±0.1%	1.3±0.1%	1.2±0.1%
COOM-terminated PLA	6.4±0.5%	4.5±0.4%	4.0±0.4%	2.9±0.2%	4.1±0.8%

SD (standard deviation) of the mean for three sample measurements.

Table S4. Average charge state (ACS) of e-L100-S and the number of the ratio of acid-terminated PLA, employed using charge manipulations (NBA: 3-nitrobenzyl alcohol, as a supercharging reagent, TEA: Triethylamine, as a charge reduction reagent)

	NaI	CsI	NaI+NBA	TEA
ACS Mean±SD	1.30±0.02	1.05±0.004	1.57±0.01	1.01±0.005
N_{acid} Mean±SD	24.0±0.6%	5.3±0.8%	28.7±0.1%	19.4±2.0%

SD (standard deviation) of the mean for three measurements of each sample.

Results from chemical degradation of PLGA

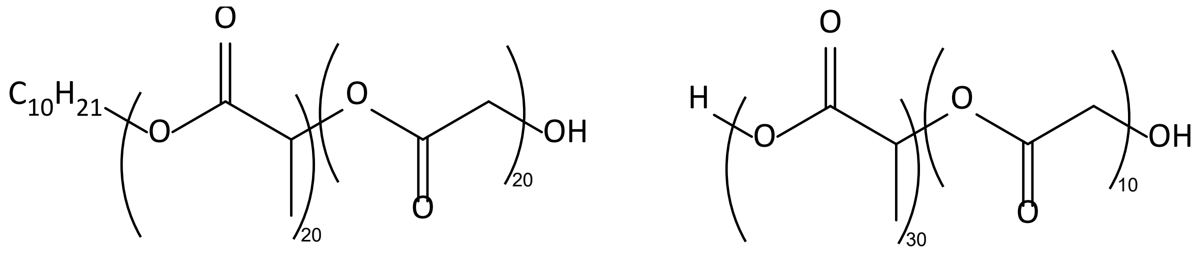

Alkyl-terminated PLGA

(LA/GA=50/50, $C_{110}H_{142}O_{81}$)

Acid-terminated PLGA

(LA/GA=75/25, $C_{110}H_{142}O_{81}$)

Figure S18. An example of isomers with the same molar mass between alkyl-terminated and acid-terminated PLGA (The examples are alkyl-terminated PLGA with LA/GA=50/50 and acid-terminated PLGA with LA/GA=75/25)

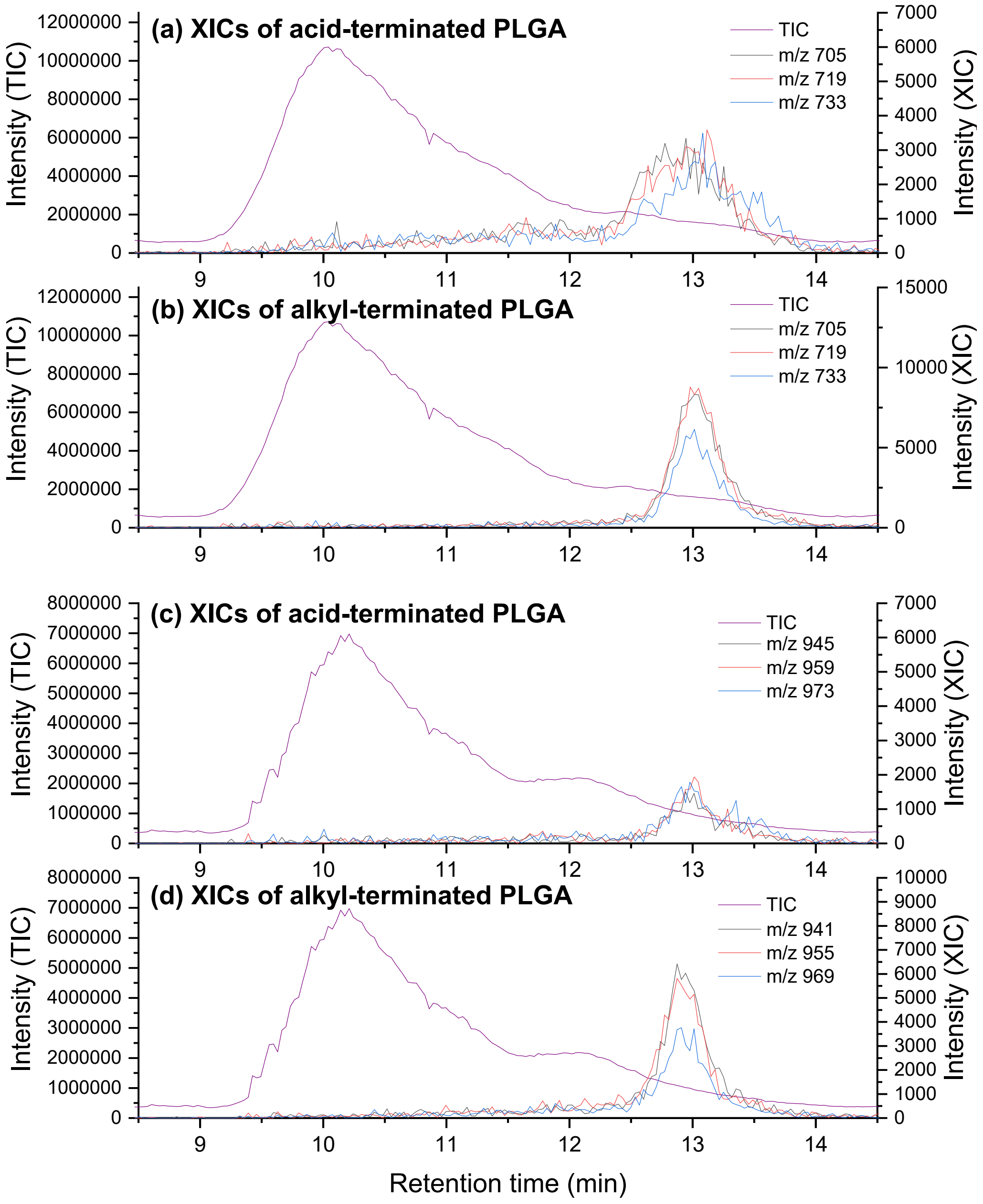

Figure S19. SEC-MS analysis of e-L50-S showing XICs of selected oligomers with alkyl (a) and acid (b) end group using NaI as a cation additive and these with alkyl (c) and acid (d) end group using CsI as a cation additive.

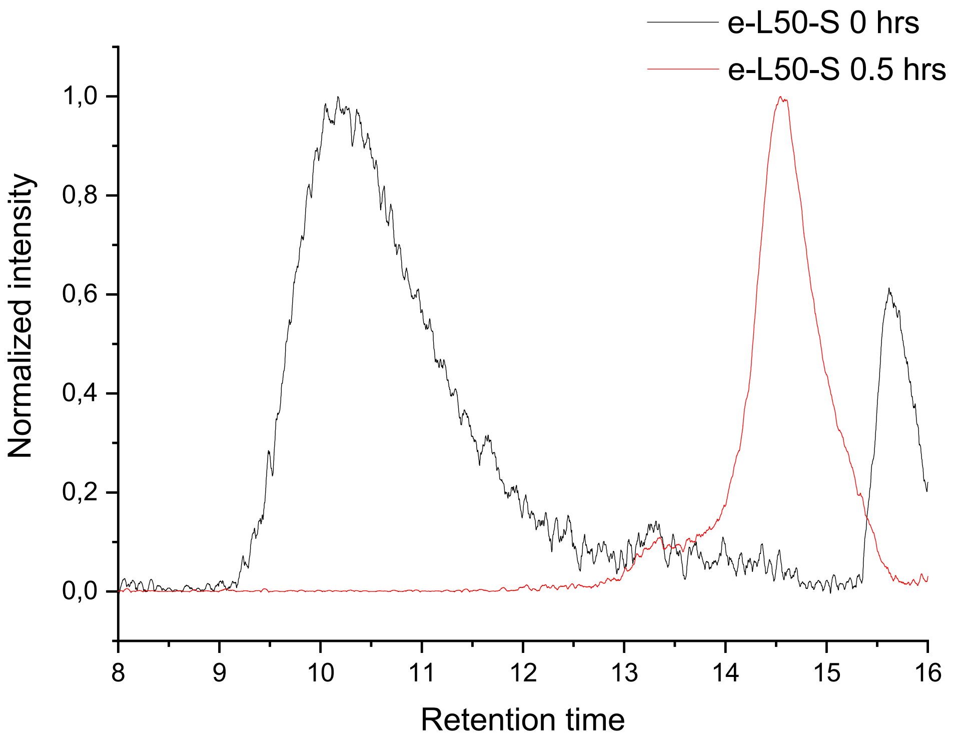

Figure S20. SEC-UV chromatograms (UV 220 nm) of e-L50-S before degradation and that of e-L50-S after 0.5 hrs degradation

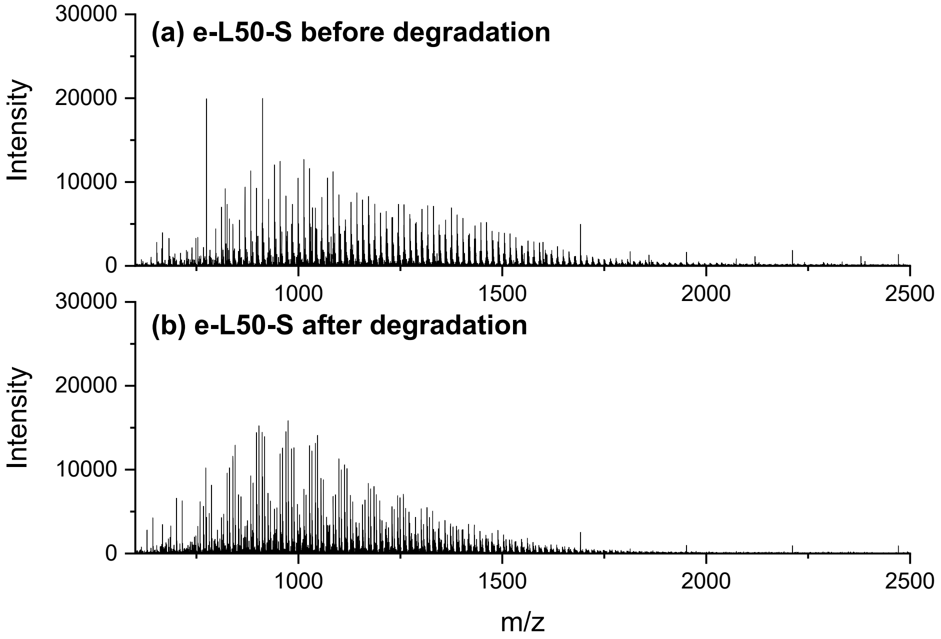

Figure S21. (a) MS spectra of e-L50-S before degradation extracted between 12.5 and 13.0 minutes of SEC-MS analysis and (b) that of e-L50-S after 0.5 hrs degradation extracted between 12.5 and 13.0 minutes of SEC-MS analysis

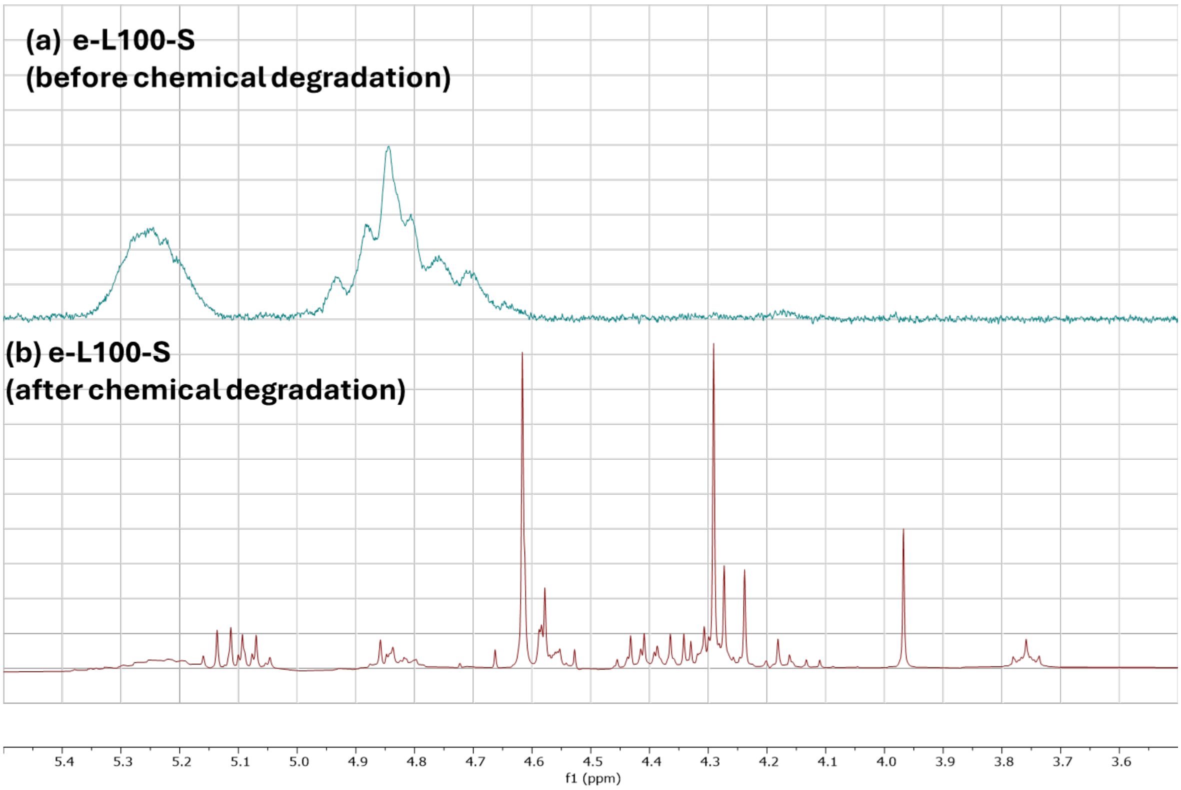

Figure S22. ^{1}H-NMR (300 MHz, Chroloform-D_1) spectra of e-L50-S before and after chemical degradation, respectively (a and b)

Table S5. Polystyrene equivalent molar masses and polydispersity of PLGAs shown in Figure S20

Abbreviation	Degradation time (hrs)	Mn (*kDa*)	Mw (*kDa*)	PDI
e-L50-S	0	12.1	28.7	2.37
e-L50-S	0.5	1.0	1.4	1.40

Table S6. Accurate mass measurements of product ions detected in MS spectra of e-L50-S before degradation extracted between 12.5 and 13.0 minutes of SEC-MS analysis

MF	Adduct	MF mass	m/z	Exp m/z	Δ ppm	Intensity	End group	LA unit number	GA unit number
C50H60O40	(Cs+)	1300.2661	1433.1710	1433.1748	2.68	0.38	Cyclic	10	10
C52H62O42	(Cs+)	1358.2716	1491.1765	1491.1800	2.37	0.31	Cyclic	10	11
C54H64O44	(Cs+)	1416.2770	1549.1819	1549.1858	2.49	0.18	Cyclic	10	12
C42H52O32	(Cs+)	1068.2442	1201.1491	1201.1517	2.21	0.47	Cyclic	10	6
C44H54O34	(Cs+)	1126.2496	1259.1546	1259.1562	1.35	0.57	Cyclic	10	7
C46H56O36	(Cs+)	1184.2551	1317.1600	1317.1595	-0.42	0.57	Cyclic	10	8
C48H58O38	(Cs+)	1242.2606	1375.1655	1375.1695	2.87	0.53	Cyclic	10	9
C53H64O42	(Cs+)	1372.2872	1505.1921	1505.1921	-0.05	0.31	Cyclic	11	10
C55H66O44	(Cs+)	1430.2927	1563.1976	1563.2011	2.27	0.23	Cyclic	11	11
C47H58O36	(Cs+)	1198.2708	1331.1757	1331.1792	2.67	0.54	Cyclic	11	7
C49H60O38	(Cs+)	1256.2763	1389.1812	1389.1782	-2.15	0.47	Cyclic	11	8
C51H62O40	(Cs+)	1314.2817	1447.1866	1447.1844	-1.58	0.40	Cyclic	11	9
C56H68O44	(Cs+)	1444.3083	1577.2132	1577.2163	1.91	0.22	Cyclic	12	10
C58H70O46	(Cs+)	1502.3138	1635.2187	1635.2189	0.10	0.18	Cyclic	12	11
C44H56O32	(Cs+)	1096.2755	1229.1804	1229.1851	3.85	0.20	Cyclic	12	4
C46H58O34	(Cs+)	1154.2809	1287.1859	1287.1901	3.33	0.33	Cyclic	12	5
C48H60O36	(Cs+)	1212.2864	1345.1913	1345.1950	2.75	0.37	Cyclic	12	6
C50H62O38	(Cs+)	1270.2919	1403.1968	1403.1971	0.20	0.44	Cyclic	12	7
C52H64O40	(Cs+)	1328.2974	1461.2023	1461.2008	-1.03	0.41	Cyclic	12	8
C54H66O42	(Cs+)	1386.3029	1519.2078	1519.2071	-0.43	0.29	Cyclic	12	9
C51H64O38	(Cs+)	1284.3076	1417.2125	1417.2092	-2.33	0.28	Cyclic	13	6
C53H66O40	(Cs+)	1342.3130	1475.2179	1475.2207	1.86	0.32	Cyclic	13	7
C55H68O42	(Cs+)	1400.3185	1533.2234	1533.2235	0.08	0.26	Cyclic	13	8
C57H70O44	(Cs+)	1458.3240	1591.2289	1591.2231	-3.61	0.21	Cyclic	13	9
C52H66O38	(Cs+)	1298.3232	1431.2281	1431.2337	3.87	0.19	Cyclic	14	5
C54H68O40	(Cs+)	1356.3287	1489.2336	1489.2370	2.27	0.22	Cyclic	14	6
C56H70O42	(Cs+)	1414.3342	1547.2391	1547.2390	-0.06	0.18	Cyclic	14	7
C38H44O32	(Cs+)	1012.1816	1145.0865	1145.0902	3.29	0.23	Cyclic	6	10
C40H46O34	(Cs+)	1070.1870	1203.0920	1203.0967	3.93	0.19	Cyclic	6	11
C34H40O28	(Cs+)	896.1706	1029.0755	1029.0749	-0.58	0.22	Cyclic	6	8
C36H42O30	(Cs+)	954.1761	1087.0810	1087.0818	0.71	0.21	Cyclic	6	9
C41H48O34	(Cs+)	1084.2027	1217.1076	1217.1110	2.76	0.34	Cyclic	7	10
C43H50O36	(Cs+)	1142.2082	1275.1131	1275.1102	-2.30	0.23	Cyclic	7	11
C45H52O38	(Cs+)	1200.2137	1333.1186	1333.1326	10.56	0.19	Cyclic	7	12
C33H40O26	(Cs+)	852.1808	985.0857	985.0875	1.87	0.15	Cyclic	7	6
C35H42O28	(Cs+)	910.1863	1043.0912	1043.0949	3.61	0.31	Cyclic	7	7
C37H44O30	(Cs+)	968.1917	1101.0966	1101.0993	2.44	0.37	Cyclic	7	8
C39H46O32	(Cs+)	1026.1972	1159.1021	1159.1082	5.20	0.38	Cyclic	7	9
C44H52O36	(Cs+)	1156.2238	1289.1287	1289.1319	2.44	0.41	Cyclic	8	10
C46H54O38	(Cs+)	1214.2293	1347.1342	1347.1338	-0.33	0.28	Cyclic	8	11
C48H56O40	(Cs+)	1272.2348	1405.1397	1405.1377	-1.39	0.22	Cyclic	8	12
C36H44O28	(Cs+)	924.2019	1057.1068	1057.1038	-2.89	0.33	Cyclic	8	6
C38H46O30	(Cs+)	982.2074	1115.1123	1115.1118	-0.47	0.42	Cyclic	8	7

C40H48O32	(Cs+)	1040.2129	1173.1178	1173.1225	4.04	0.47	Cyclic	8	8
C42H50O34	(Cs+)	1098.2183	1231.1233	1231.1224	-0.67	0.46	Cyclic	8	9
C47H56O38	(Cs+)	1228.2450	1361.1499	1361.1511	0.90	0.42	Cyclic	9	10
C49H58O40	(Cs+)	1286.2504	1419.1553	1419.1593	2.77	0.29	Cyclic	9	11
C51H60O42	(Cs+)	1344.2559	1477.1608	1477.1675	4.50	0.23	Cyclic	9	12
C37H46O28	(Cs+)	938.2176	1071.1225	1071.1243	1.70	0.27	Cyclic	9	5
C39H48O30	(Cs+)	996.2230	1129.1279	1129.1274	-0.49	0.44	Cyclic	9	6
C41H50O32	(Cs+)	1054.2285	1187.1334	1187.1411	6.46	0.59	Cyclic	9	7
C43H52O34	(Cs+)	1112.2340	1245.1389	1245.1382	-0.53	0.56	Cyclic	9	8
C45H54O36	(Cs+)	1170.2395	1303.1444	1303.1496	3.97	0.53	Cyclic	9	9
C34H46O25	(Cs+)	854.2328	987.1377	987.1407	3.02	0.21	Alkyl end group	0	12
C36H48O27	(Cs+)	912.2383	1045.1432	1045.1418	-1.37	0.33	Alkyl end group	0	13
C38H50O29	(Cs+)	970.2438	1103.1487	1103.1480	-0.64	0.29	Alkyl end group	0	14
C40H52O31	(Cs+)	1028.2493	1161.1542	1161.1541	-0.09	0.23	Alkyl end group	0	15
C42H54O33	(Cs+)	1086.2547	1219.1596	1219.1564	-2.67	0.21	Alkyl end group	0	16
C39H52O29	(Cs+)	984.2594	1117.1643	1117.1684	3.61	0.21	Alkyl end group	1	13
C41H54O31	(Cs+)	1042.2649	1175.1698	1175.1695	-0.27	0.21	Alkyl end group	1	14
C43H56O33	(Cs+)	1100.2704	1233.1753	1233.1616	-11.07	0.20	Alkyl end group	1	15
C44H66O25	(Cs+)	994.3893	1127.2942	1127.2892	-4.47	0.15	Alkyl end group	10	2
C46H68O27	(Cs+)	1052.3948	1185.2997	1185.2983	-1.20	0.23	Alkyl end group	10	3
C48H70O29	(Cs+)	1110.4003	1243.3052	1243.3030	-1.75	0.30	Alkyl end group	10	4
C50H72O31	(Cs+)	1168.4058	1301.3107	1301.3091	-1.21	0.31	Alkyl end group	10	5
C52H74O33	(Cs+)	1226.4112	1359.3161	1359.3181	1.44	0.28	Alkyl end group	10	6
C54H76O35	(Cs+)	1284.4167	1417.3216	1417.3171	-3.18	0.24	Alkyl end group	10	7
C51H74O31	(Cs+)	1182.4214	1315.3263	1315.3243	-1.56	0.19	Alkyl end group	11	4
C53H76O33	(Cs+)	1240.4269	1373.3318	1373.3392	5.41	0.17	Alkyl end group	11	5
C55H78O35	(Cs+)	1298.4324	1431.3373	1431.3411	2.68	0.20	Alkyl end group	11	6
C57H80O37	(Cs+)	1356.4378	1489.3427	1489.3452	1.66	0.16	Alkyl end group	11	7
C42H56O31	(Cs+)	1056.2806	1189.1855	1189.1807	-3.98	0.15	Alkyl end group	2	13
C27H42O15	(Cs+)	606.2524	739.1573	739.1579	0.84	0.17	Alkyl end group	3	4
C29H44O17	(Cs+)	664.2578	797.1628	797.1644	2.01	0.35	Alkyl end group	3	5
C31H46O19	(Cs+)	722.2633	855.1682	855.1682	-0.02	0.43	Alkyl end group	3	6
C33H48O21	(Cs+)	780.2688	913.1737	913.1753	1.77	0.35	Alkyl end group	3	7
C35H50O23	(Cs+)	838.2743	971.1792	971.1809	1.75	0.22	Alkyl end group	3	8
C28H44O15	(Cs+)	620.2680	753.1729	753.1739	1.33	0.26	Alkyl end group	4	3
C30H46O17	(Cs+)	678.2735	811.1784	811.1800	1.92	0.55	Alkyl end group	4	4
C32H48O19	(Cs+)	736.2790	869.1839	869.1859	2.27	0.72	Alkyl end group	4	5

C34H50O21	(Cs+)	794.2845	927.1894	927.1891	-0.26	0.61	Alkyl end group	4	6
C36H52O23	(Cs+)	852.2899	985.1948	985.1956	0.81	0.56	Alkyl end group	4	7
C38H54O25	(Cs+)	910.2954	1043.2003	1043.1985	-1.76	0.34	Alkyl end group	4	8
C29H46O15	(Cs+)	634.2837	767.1886	767.1874	-1.57	0.17	Alkyl end group	5	2
C31H48O17	(Cs+)	692.2892	825.1941	825.1952	1.43	0.57	Alkyl end group	5	3
C33H50O19	(Cs+)	750.2946	883.1995	883.2000	0.54	0.87	Alkyl end group	5	4
C35H52O21	(Cs+)	808.3001	941.2050	941.2063	1.34	0.94	Alkyl end group	5	5
C37H54O23	(Cs+)	866.3056	999.2105	999.2104	-0.05	0.81	Alkyl end group	5	6
C39H56O25	(Cs+)	924.3111	1057.2160	1057.2157	-0.27	0.62	Alkyl end group	5	7
C41H58O27	(Cs+)	982.3165	1115.2214	1115.2163	-4.65	0.36	Alkyl end group	5	8
C43H60O29	(Cs+)	1040.3220	1173.2269	1173.2253	-1.35	0.20	Alkyl end group	5	9
C48H66O33	(Cs+)	1170.3486	1303.2535	1303.2600	4.94	0.17	Alkyl end group	6	10
C32H50O17	(Cs+)	706.3048	839.2097	839.2097	-0.05	0.38	Alkyl end group	6	2
C34H52O19	(Cs+)	764.3103	897.2152	897.2141	-1.25	0.71	Alkyl end group	6	3
C36H54O21	(Cs+)	822.3158	955.2207	955.2218	1.20	0.96	Alkyl end group	6	4
C38H56O23	(Cs+)	880.3212	1013.2261	1013.2234	-2.70	1.00	Alkyl end group	6	5
C40H58O25	(Cs+)	938.3267	1071.2316	1071.2304	-1.14	0.81	Alkyl end group	6	6
C42H60O27	(Cs+)	996.3322	1129.2371	1129.2369	-0.19	0.58	Alkyl end group	6	7
C44H62O29	(Cs+)	1054.3377	1187.2426	1187.2438	1.05	0.40	Alkyl end group	6	8
C46H64O31	(Cs+)	1112.3432	1245.2481	1245.2543	4.99	0.26	Alkyl end group	6	9
C51H70O35	(Cs+)	1242.3698	1375.2747	1375.2782	2.57	0.15	Alkyl end group	7	10
C35H54O19	(Cs+)	778.3259	911.2308	911.2315	0.72	0.39	Alkyl end group	7	2
C37H56O21	(Cs+)	836.3314	969.2363	969.2395	3.32	0.67	Alkyl end group	7	3
C39H58O23	(Cs+)	894.3369	1027.2418	1027.2405	-1.26	0.89	Alkyl end group	7	4
C41H60O25	(Cs+)	952.3424	1085.2473	1085.2450	-2.14	0.86	Alkyl end group	7	5
C43H62O27	(Cs+)	1010.3478	1143.2528	1143.2544	1.45	0.67	Alkyl end group	7	6
C45H64O29	(Cs+)	1068.3533	1201.2582	1201.2582	0.00	0.50	Alkyl end group	7	7
C47H66O31	(Cs+)	1126.3588	1259.2637	1259.2671	2.66	0.37	Alkyl end group	7	8
C49H68O33	(Cs+)	1184.3643	1317.2692	1317.2642	-3.78	0.26	Alkyl end group	7	9
C38H58O21	(Cs+)	850.3471	983.2520	983.2512	-0.81	0.38	Alkyl end group	8	2
C40H60O23	(Cs+)	908.3525	1041.2574	1041.2572	-0.20	0.54	Alkyl end group	8	3
C42H62O25	(Cs+)	966.3580	1099.2629	1099.2626	-0.26	0.65	Alkyl end group	8	4
C44H64O27	(Cs+)	1024.3635	1157.2684	1157.2678	-0.53	0.62	Alkyl end group	8	5
C46H66O29	(Cs+)	1082.3690	1215.2739	1215.2764	2.05	0.51	Alkyl end group	8	6
C48H68O31	(Cs+)	1140.3745	1273.2794	1273.2790	-0.24	0.43	Alkyl end group	8	7

C50H70O33	(Cs+)	1198.3799	1331.2848	1331.2875	2.02	0.35	Alkyl end group	8	8
C41H62O23	(Cs+)	922.3682	1055.2731	1055.2798	6.35	0.23	Alkyl end group	9	2
C43H64O25	(Cs+)	980.3737	1113.2786	1113.2795	0.80	0.32	Alkyl end group	9	3
C45H66O27	(Cs+)	1038.3791	1171.2841	1171.2822	-1.56	0.39	Alkyl end group	9	4
C47H68O29	(Cs+)	1096.3846	1229.2895	1229.2923	2.22	0.44	Alkyl end group	9	5
C49H70O31	(Cs+)	1154.3901	1287.2950	1287.2948	-0.15	0.39	Alkyl end group	9	6
C51H72O33	(Cs+)	1212.3956	1345.3005	1345.2999	-0.45	0.33	Alkyl end group	9	7
C53H74O35	(Cs+)	1270.4011	1403.3060	1403.3040	-1.42	0.25	Alkyl end group	9	8

Table S7. Accurate mass measurements of product ions detected in MS spectra of e-L50-S after 0.5hrs degradation extracted between 12.5 and 13.0 minutes of SEC-MS analysis

MF	Adduct	MF mass	m/z	Exp m/z	Δ ppm	Intensity	End group	LA unit number	GA unit number
C42H52O32	(Cs+)	1068.2442	1201.1491	1201.1502	0.95	0.15	Cyclic	10	6
C43H54O32	(Cs+)	1082.2598	1215.1647	1215.1659	0.95	0.21	Cyclic	11	5
C45H56O34	(Cs+)	1140.2653	1273.1702	1273.1806	8.19	0.23	Cyclic	11	6
C47H58O36	(Cs+)	1198.2708	1331.1757	1331.1771	1.10	0.18	Cyclic	11	7
C44H56O32	(Cs+)	1096.2755	1229.1804	1229.1839	2.86	0.25	Cyclic	12	4
C46H58O34	(Cs+)	1154.2809	1287.1859	1287.1935	5.97	0.27	Cyclic	12	5
C48H60O36	(Cs+)	1212.2864	1345.1913	1345.1985	5.30	0.23	Cyclic	12	6
C50H62O38	(Cs+)	1270.2919	1403.1968	1403.2015	3.36	0.18	Cyclic	12	7
C49H62O36	(Cs+)	1226.3021	1359.2070	1359.2156	6.31	0.25	Cyclic	13	5
C51H64O38	(Cs+)	1284.3076	1417.2125	1417.2156	2.22	0.22	Cyclic	13	6
C53H66O40	(Cs+)	1342.3130	1475.2179	1475.2205	1.74	0.15	Cyclic	13	7
C48H62O34	(Cs+)	1182.3122	1315.2172	1315.2204	2.44	0.20	Cyclic	14	3
C50H64O36	(Cs+)	1240.3177	1373.2226	1373.2187	-2.89	0.20	Cyclic	14	4
C52H66O38	(Cs+)	1298.3232	1431.2281	1431.2298	1.18	0.21	Cyclic	14	5
C54H68O40	(Cs+)	1356.3287	1489.2336	1489.2348	0.83	0.17	Cyclic	14	6
C53H68O38	(Cs+)	1312.3389	1445.2438	1445.2421	-1.13	0.17	Cyclic	15	4
C30H34O26	(Cs+)	810.1338	943.0387	943.0433	4.87	0.15	Cyclic	4	9
C35H40O30	(Cs+)	940.1604	1073.0653	1073.0702	4.52	0.16	Cyclic	5	10
C31H36O26	(Cs+)	824.1495	957.0544	957.0579	3.65	0.16	Cyclic	5	8
C33H38O28	(Cs+)	882.1550	1015.0599	1015.0580	-1.80	0.18	Cyclic	5	9
C42H64O23	(Cs+)	936.3838	1069.2887	1069.2898	0.99	0.28	Alkyl end group	10	1
C44H66O25	(Cs+)	994.3893	1127.2942	1127.2972	2.64	0.43	Alkyl end group	10	2
C46H68O27	(Cs+)	1052.3948	1185.2997	1185.3016	1.63	0.50	Alkyl end group	10	3
C48H70O29	(Cs+)	1110.4003	1243.3052	1243.3127	6.05	0.44	Alkyl end group	10	4
C50H72O31	(Cs+)	1168.4058	1301.3107	1301.3126	1.47	0.33	Alkyl end group	10	5
C52H74O33	(Cs+)	1226.4112	1359.3161	1359.3196	2.58	0.18	Alkyl end group	10	6
C45H68O25	(Cs+)	1008.4050	1141.3099	1141.3190	7.99	0.23	Alkyl end group	11	1
C47H70O27	(Cs+)	1066.4104	1199.3154	1199.3154	0.01	0.39	Alkyl end group	11	2

C49H72O29	(Cs+)	1124.4159	1257.3208	1257.3245	2.96	0.44	Alkyl end group	11	3
C51H74O31	(Cs+)	1182.4214	1315.3263	1315.3279	1.20	0.34	Alkyl end group	11	4
C53H76O33	(Cs+)	1240.4269	1373.3318	1373.3403	6.17	0.22	Alkyl end group	11	5
C48H72O27	(Cs+)	1080.4261	1213.3310	1213.3295	-1.20	0.19	Alkyl end group	12	1
C50H74O29	(Cs+)	1138.4316	1271.3365	1271.3408	3.38	0.32	Alkyl end group	12	2
C52H76O31	(Cs+)	1196.4371	1329.3420	1329.3470	3.79	0.31	Alkyl end group	12	3
C54H78O33	(Cs+)	1254.4425	1387.3474	1387.3449	-1.83	0.21	Alkyl end group	12	4
C53H78O31	(Cs+)	1210.4527	1343.3576	1343.3644	5.05	0.19	Alkyl end group	13	2
C55H80O33	(Cs+)	1268.4582	1401.3631	1401.3696	4.68	0.17	Alkyl end group	13	3
C28H44O15	(Cs+)	620.2680	753.1729	753.1755	3.40	0.20	Alkyl end group	4	3
C30H46O17	(Cs+)	678.2735	811.1784	811.1801	2.09	0.27	Alkyl end group	4	4
C32H48O19	(Cs+)	736.2790	869.1839	869.1877	4.34	0.20	Alkyl end group	4	5
C29H46O15	(Cs+)	634.2837	767.1886	767.1907	2.81	0.35	Alkyl end group	5	2
C31H48O17	(Cs+)	692.2892	825.1941	825.1962	2.55	0.60	Alkyl end group	5	3
C33H50O19	(Cs+)	750.2946	883.1995	883.2007	1.27	0.57	Alkyl end group	5	4
C35H52O21	(Cs+)	808.3001	941.2050	941.2066	1.68	0.34	Alkyl end group	5	5
C37H54O23	(Cs+)	866.3056	999.2105	999.2152	4.73	0.17	Alkyl end group	5	6
C30H48O15	(Cs+)	648.2993	781.2042	781.2073	3.94	0.29	Alkyl end group	6	1
C32H50O17	(Cs+)	706.3048	839.2097	839.2117	2.35	0.74	Alkyl end group	6	2
C34H52O19	(Cs+)	764.3103	897.2152	897.2171	2.16	0.89	Alkyl end group	6	3
C36H54O21	(Cs+)	822.3158	955.2207	955.2228	2.27	0.75	Alkyl end group	6	4
C38H56O23	(Cs+)	880.3212	1013.2261	1013.2310	4.77	0.47	Alkyl end group	6	5
C40H58O25	(Cs+)	938.3267	1071.2316	1071.2347	2.86	0.21	Alkyl end group	6	6
C33H52O17	(Cs+)	720.3205	853.2254	853.2302	5.68	0.45	Alkyl end group	7	1
C35H54O19	(Cs+)	778.3259	911.2308	911.2332	2.60	0.89	Alkyl end group	7	2
C37H56O21	(Cs+)	836.3314	969.2363	969.2384	2.17	0.93	Alkyl end group	7	3
C39H58O23	(Cs+)	894.3369	1027.2418	1027.2443	2.43	0.79	Alkyl end group	7	4
C41H60O25	(Cs+)	952.3424	1085.2473	1085.2481	0.75	0.43	Alkyl end group	7	5
C43H62O27	(Cs+)	1010.3478	1143.2528	1143.2555	2.44	0.19	Alkyl end group	7	6
C36H56O19	(Cs+)	792.3416	925.2465	925.2487	2.40	0.45	Alkyl end group	8	1
C38H58O21	(Cs+)	850.3471	983.2520	983.2558	3.88	0.78	Alkyl end group	8	2
C40H60O23	(Cs+)	908.3525	1041.2574	1041.2597	2.18	0.84	Alkyl end group	8	3
C42H62O25	(Cs+)	966.3580	1099.2629	1099.2656	2.46	0.70	Alkyl end group	8	4
C44H64O27	(Cs+)	1024.3635	1157.2684	1157.2699	1.28	0.36	Alkyl end group	8	5
C46H66O29	(Cs+)	1082.3690	1215.2739	1215.2793	4.46	0.24	Alkyl end group	8	6

C39H60O21	(Cs+)	864.3627	997.2676	997.2690	1.35	0.37	Alkyl end group	9	1
C41H62O23	(Cs+)	922.3682	1055.2731	1055.2751	1.92	0.56	Alkyl end group	9	2
C43H64O25	(Cs+)	980.3737	1113.2786	1113.2794	0.76	0.67	Alkyl end group	9	3
C45H66O27	(Cs+)	1038.3791	1171.2841	1171.2880	3.34	0.52	Alkyl end group	9	4
C47H68O29	(Cs+)	1096.3846	1229.2895	1229.2947	4.23	0.35	Alkyl end group	9	5
C49H70O31	(Cs+)	1154.3901	1287.2950	1287.2997	3.68	0.24	Alkyl end group	9	6
C32H43CsO23	(Cs+)	928.1250	1061.0299	1061.0300	0.09	0.56	Acid end group	10	1
C34H45CsO25	(Cs+)	986.1304	1119.0353	1119.0353	-0.05	0.63	Acid end group	10	2
C36H47CsO27	(Cs+)	1044.1359	1177.0408	1177.0432	2.00	0.48	Acid end group	10	3
C38H49CsO29	(Cs+)	1102.1414	1235.0463	1235.0531	5.48	0.33	Acid end group	10	4
C30H41CsO21	(Cs+)	870.1195	1003.0244	1003.0267	2.31	0.29	Acid end group	10	0
C35H47CsO25	(Cs+)	1000.1461	1133.0510	1133.0520	0.85	0.38	Acid end group	11	1
C37H49CsO27	(Cs+)	1058.1516	1191.0565	1191.0615	4.22	0.43	Acid end group	11	2
C39H51CsO29	(Cs+)	1116.1571	1249.0620	1249.0666	3.73	0.41	Acid end group	11	3
C41H53CsO31	(Cs+)	1174.1625	1307.0674	1307.0695	1.61	0.22	Acid end group	11	4
C33H45CsO23	(Cs+)	942.1406	1075.0455	1075.0497	3.86	0.21	Acid end group	11	0
C38H51CsO27	(Cs+)	1072.1672	1205.0721	1205.0770	4.08	0.25	Acid end group	12	1
C40H53CsO29	(Cs+)	1130.1727	1263.0776	1263.0828	4.10	0.33	Acid end group	12	2
C42H55CsO31	(Cs+)	1188.1782	1321.0831	1321.0876	3.42	0.27	Acid end group	12	3
C44H57CsO33	(Cs+)	1246.1837	1379.0886	1379.0925	2.83	0.20	Acid end group	12	4
C43H57CsO31	(Cs+)	1202.1938	1335.0987	1335.1032	3.38	0.16	Acid end group	13	2
C45H59CsO33	(Cs+)	1260.1993	1393.1042	1393.1053	0.79	0.18	Acid end group	13	3
C14H19CsO11	(Cs+)	495.9982	628.9031	628.9073	6.68	0.17	Acid end group	4	1
C16H21CsO13	(Cs+)	554.0037	686.9086	686.9145	8.64	0.21	Acid end group	4	2
C17H23CsO13	(Cs+)	568.0193	700.9242	700.9288	6.53	0.41	Acid end group	5	1
C19H25CsO15	(Cs+)	626.0248	758.9297	758.9330	4.40	0.38	Acid end group	5	2
C21H27CsO17	(Cs+)	684.0303	816.9352	816.9396	5.42	0.29	Acid end group	5	3
C23H29CsO19	(Cs+)	742.0358	874.9407	874.9467	6.89	0.24	Acid end group	5	4
C15H21CsO11	(Cs+)	510.0138	642.9187	642.9225	5.79	0.27	Acid end group	5	0
C20H27CsO15	(Cs+)	640.0404	772.9454	772.9471	2.28	0.63	Acid end group	6	1
C22H29CsO17	(Cs+)	698.0459	830.9508	830.9524	1.86	0.65	Acid end group	6	2
C24H31CsO19	(Cs+)	756.0514	888.9563	888.9585	2.45	0.52	Acid end group	6	3
C26H33CsO21	(Cs+)	814.0569	946.9618	946.9627	1.01	0.34	Acid end group	6	4
C28H35CsO23	(Cs+)	872.0624	1004.9673	1004.9688	1.56	0.19	Acid end group	6	5
C18H25CsO13	(Cs+)	582.0350	714.9399	714.9448	6.86	0.39	Acid end group	6	0

C23H31CsO17	(Cs+)	712.0616	844.9665	844.9693	3.33	0.80	Acid end group	7	1
C25H33CsO19	(Cs+)	770.0671	902.9720	902.9738	2.03	0.95	Acid end group	7	2
C27H35CsO21	(Cs+)	828.0725	960.9774	960.9785	1.10	0.79	Acid end group	7	3
C29H37CsO23	(Cs+)	886.0780	1018.9829	1018.9869	3.91	0.44	Acid end group	7	4
C31H39CsO25	(Cs+)	944.0835	1076.9884	1076.9937	4.92	0.21	Acid end group	7	5
C21H29CsO15	(Cs+)	654.0561	786.9610	786.9632	2.77	0.50	Acid end group	7	0
C26H35CsO19	(Cs+)	784.0827	916.9876	916.9902	2.82	0.87	Acid end group	8	1
C28H37CsO21	(Cs+)	842.0882	974.9931	974.9934	0.32	1.00	Acid end group	8	2
C30H39CsO23	(Cs+)	900.0937	1032.9986	1032.9982	-0.31	0.76	Acid end group	8	3
C32H41CsO25	(Cs+)	958.0991	1091.0040	1091.0062	2.01	0.44	Acid end group	8	4
C34H43CsO27	(Cs+)	1016.1046	1149.0095	1149.0085	-0.90	0.23	Acid end group	8	5
C24H33CsO17	(Cs+)	726.0772	858.9821	858.9845	2.77	0.42	Acid end group	8	0
C29H39CsO21	(Cs+)	856.1038	989.0087	989.0120	3.27	0.79	Acid end group	9	1
C31H41CsO23	(Cs+)	914.1093	1047.0142	1047.0180	3.66	0.88	Acid end group	9	2
C33H43CsO25	(Cs+)	972.1148	1105.0197	1105.0250	4.77	0.65	Acid end group	9	3
C35H45CsO27	(Cs+)	1030.1203	1163.0252	1163.0254	0.17	0.40	Acid end group	9	4
C37H47CsO29	(Cs+)	1088.1258	1221.0307	1221.0358	4.25	0.20	Acid end group	9	5
C27H37CsO19	(Cs+)	798.0984	931.0033	931.0058	2.75	0.40	Acid end group	9	0

Table S8. PLGA contents with different end groups from chemical composition characterization shown in Figure 7

	Cyclic MS%	Alkyl MS%	Acid MS%	Overall MS%
e-L50-S 0hr	37.8%	62.2%	N.D.	100%
e-L50-S 0.5hr	8.6%	46.0%	45.4%	100%
